# Supplementary material for: B3galt5 functions as a PXR target gene and regulates obesity and insulin resistance by maintaining intestinal integrity
Source: Nat Commun. 2024 Jul 14;15:5919. doi: 10.1038/s41467-024-50198-z (PMC11247088; doi:10.1038/s41467-024-50198-z)
Supplement: Supplementary file 1 — Supplementary Information [file 41467_2024_50198_MOESM1_ESM.pdf]

Supplementary information

B3galt5 functions as a PXR target gene and regulates obesity and insulin resistance by maintaining intestinal integrity

Jinhang Zhang[1†], Ya Huang[1, 2†], Hong Li[1], Pengfei Xu[3], Qinhui Liu[1], Yang Sun[4], Zijing Zhang[1], Tong Wu[1], Qin Tang[1], Qingyi Jia[1], Yan Xia[1], Ying Xu[1], Xiandan Jing[1], Jiahui Li[1], Li Mo[5], Wen Xie[3], Aijuan Qu[6], Jinhan He[1*], and Yanping Li[1*]

†, These authors contributed equally to this paper.

[1] Department of Pharmacy, Institute of Metabolic Diseases and Pharmacotherapy, National Clinical Research Center for Geriatrics, West China Hospital, Sichuan University, Chengdu, Sichuan Province, China. [2] Department of Pharmacy, GuiQian International General Hospital, Guiyang, China. [3] Center for Pharmacogenetics and Department of Pharmaceutical Sciences, University of Pittsburgh, Pittsburgh, Pennsylvania, USA. [4] Department of Gastroenterology, The First Affiliated Hospital of Kunming Medical University, Yunnan Institute of Digestive Disease, Kunming, Yunnan Province, China.[5] Center of Gerontology and Geriatrics, West China Hospital of Sichuan University, Chengdu, China. [6] Department of Physiology and Pathophysiology, School of Basic Medical Sciences, Capital Medical University, Beijing, P.R. China.

*, Corresponding author:

Jinhan He, Department of Pharmacy, Institute of Metabolic Diseases and Pharmacotherapy, West China Hospital, Sichuan University, Chengdu, Sichuan Province, China. Tel: 86-28-85426416, Email: jinhanhe@scu.edu.cn; or Yanping Li, Department of Pharmacy, Institute of Metabolic Diseases and Pharmacotherapy, West China Hospital, Sichuan University, Chengdu, Sichuan Province, China. Tel: 86-28-85164128, Email: liyanping_512@163.com.

Supplementary figures

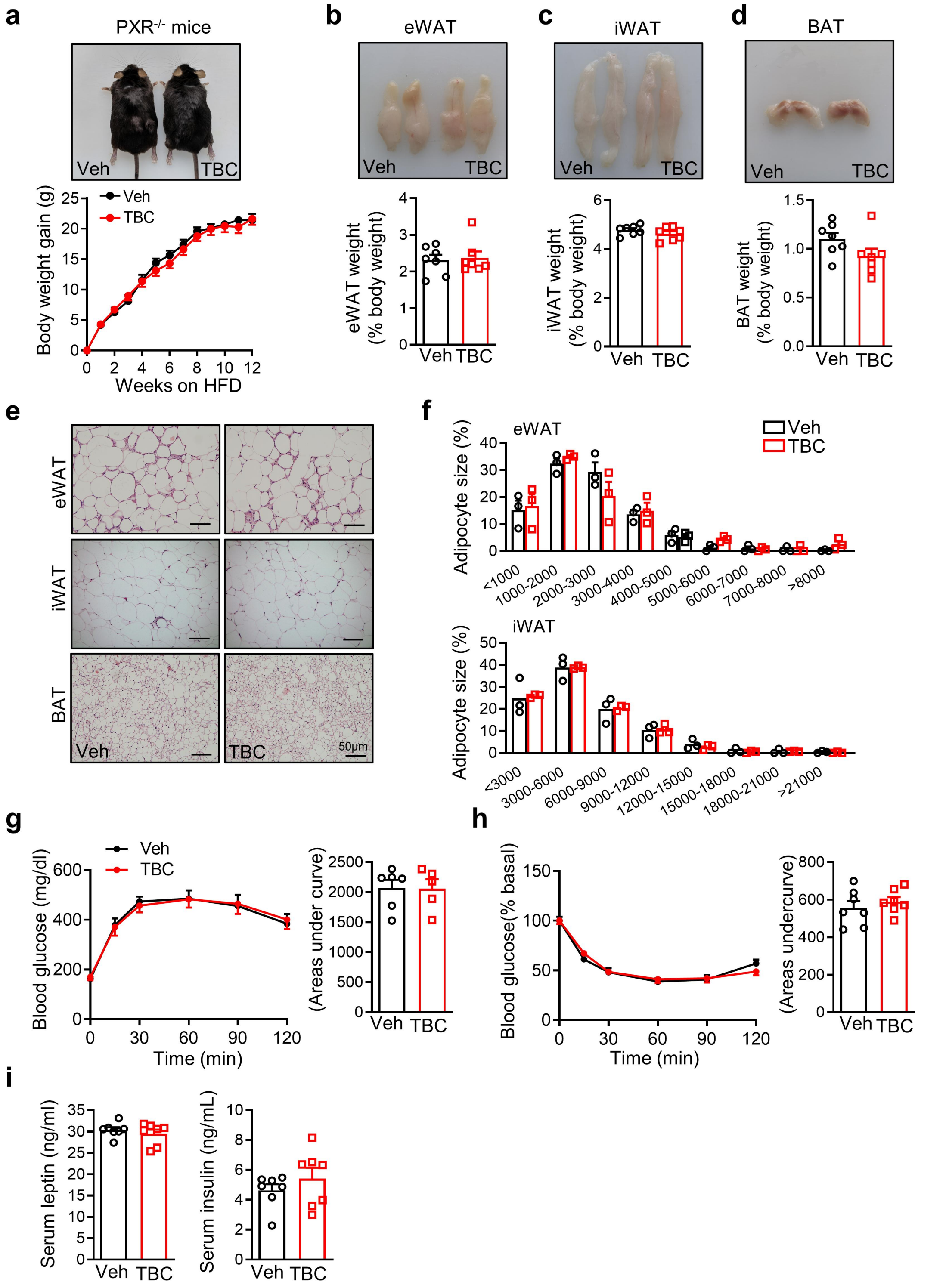

Figure S1. TBC is a PXR intestinal specific agonist. PXR KO mice were fed with TBC supplemented in HFD at 0.05% (w/w) for 12 weeks. (a) Appearance (top) and growth curve (bottom) of vehicle and TBC treated PXR KO mice (*n* = 7 per group). (b-d) Representative photographs and the ratio of fat depots to body weight of eWAT

(b), iWAT (c) and BAT (d) (*n* = 7 per group). (e-f) H&E staining of adipose tissues (e) and distribution of adipocyte size of eWAT and iWAT (f; *n* = 3 per group). Scale bar: 50 μm. (g-h) Blood glucose concentrations during GTT (1 g/kg; g; *n* = 6 for PXR-/- + Veh, *n* = 5 for PXR-/- + TBC) and ITT (1.5 U/kg; h; *n* = 7 per group) in vehicle and TBC-treated PXR KO mice. (i) Serum leptin and insulin levels in vehicle and TBC treated PXR KO mice (*n* = 7 per group). PXR-/-: PXR whole-body knockout mice; eWAT: epididymal white adipose tissue; iWAT: inguinal white adipose tissue; BAT: brown adipose tissue. Data are mean ± SEM. At least three independent experiments were repeated with similar results. Source data are provided as a Source Data file.

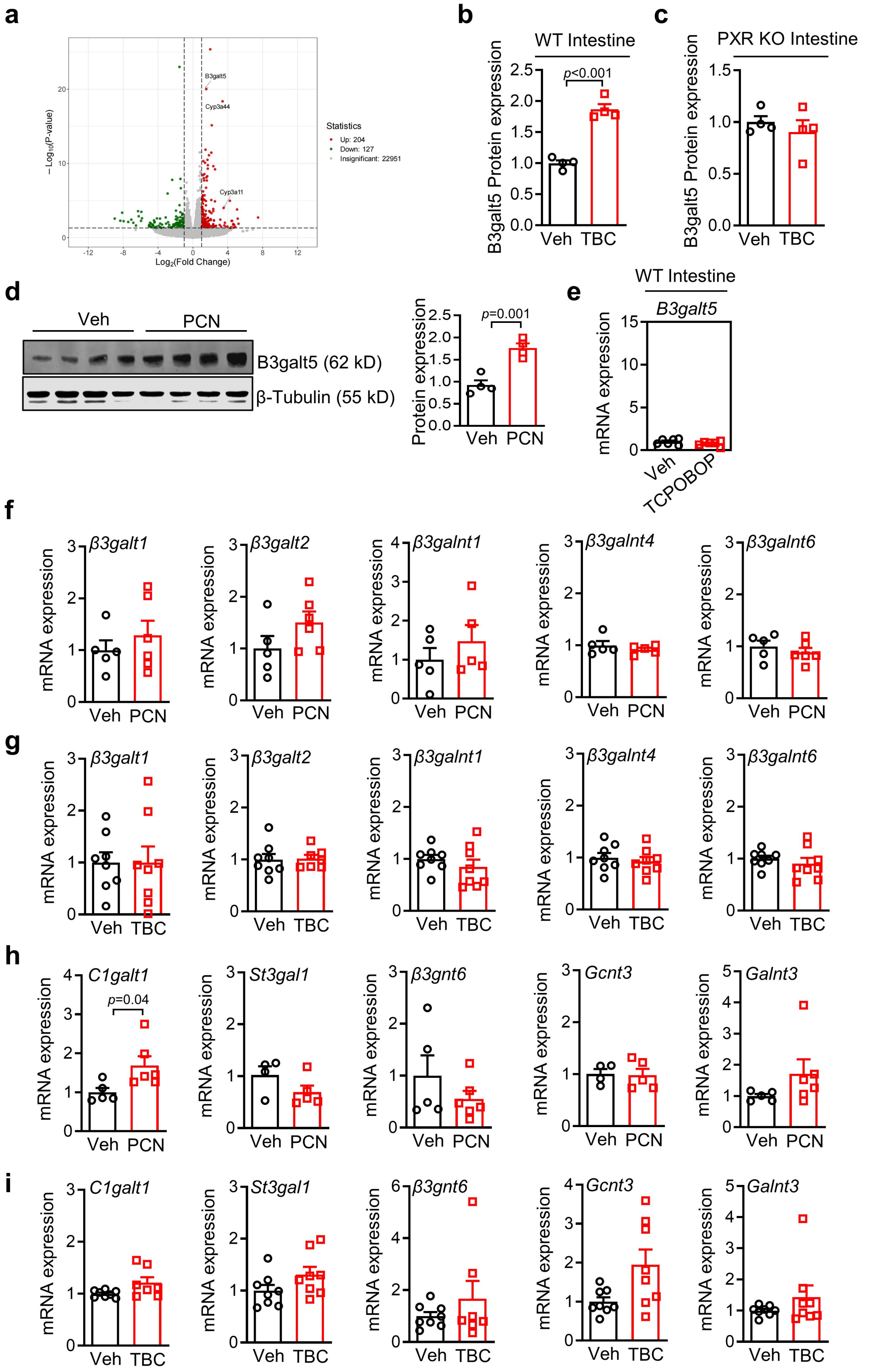

Figure S2. Activation of PXR does not influence other β3galt families and *O*-

glycosyltransferase genes. (a) The volcano plot genes changed in the colon of 8-week-old mice treated with vehicle (Veh) and PCN. Mice were treated with PCN (40 mg/kg) once every 8 hours for three times (n = 7 per group). (b-c) Semi-quantitative analysis of Figure 2d (n = 4 per group). (d) Protein levels of intestinal B3galt5 in mice treated with PCN (n = 4 per group). (e) The intestinal mRNA expression of B3galt5 in wild-type mice treated with CAR agonist TCPOBOP (10mg/kg) once every three days for two times (n = 6 per group). (f) The mRNA expression of β3galt family genes in the intestine of vehicle and PCN treated mice (n = 5 for Veh, n = 6 for PCN). (g) The mRNA expression of β3galt family genes in the intestine of vehicle and TBC treated mice (n = 8 per group). (h) The mRNA expression of *O*-glycosyltransferase genes in the intestine of vehicle and PCN treated mice (n = 5 for Veh, n = 6 for PCN). (i) The mRNA expression of *O*-glycosyltransferase genes in the intestine of vehicle and TBC treated mice (n = 8 per group). WT: wild type; PXR KO: PXR whole-body knockout mice. Data are mean ± SEM. Significance was determined using unpaired two-tailed Student's t test. At least three independent experiments were repeated with similar results. Source data are provided as a Source Data file.

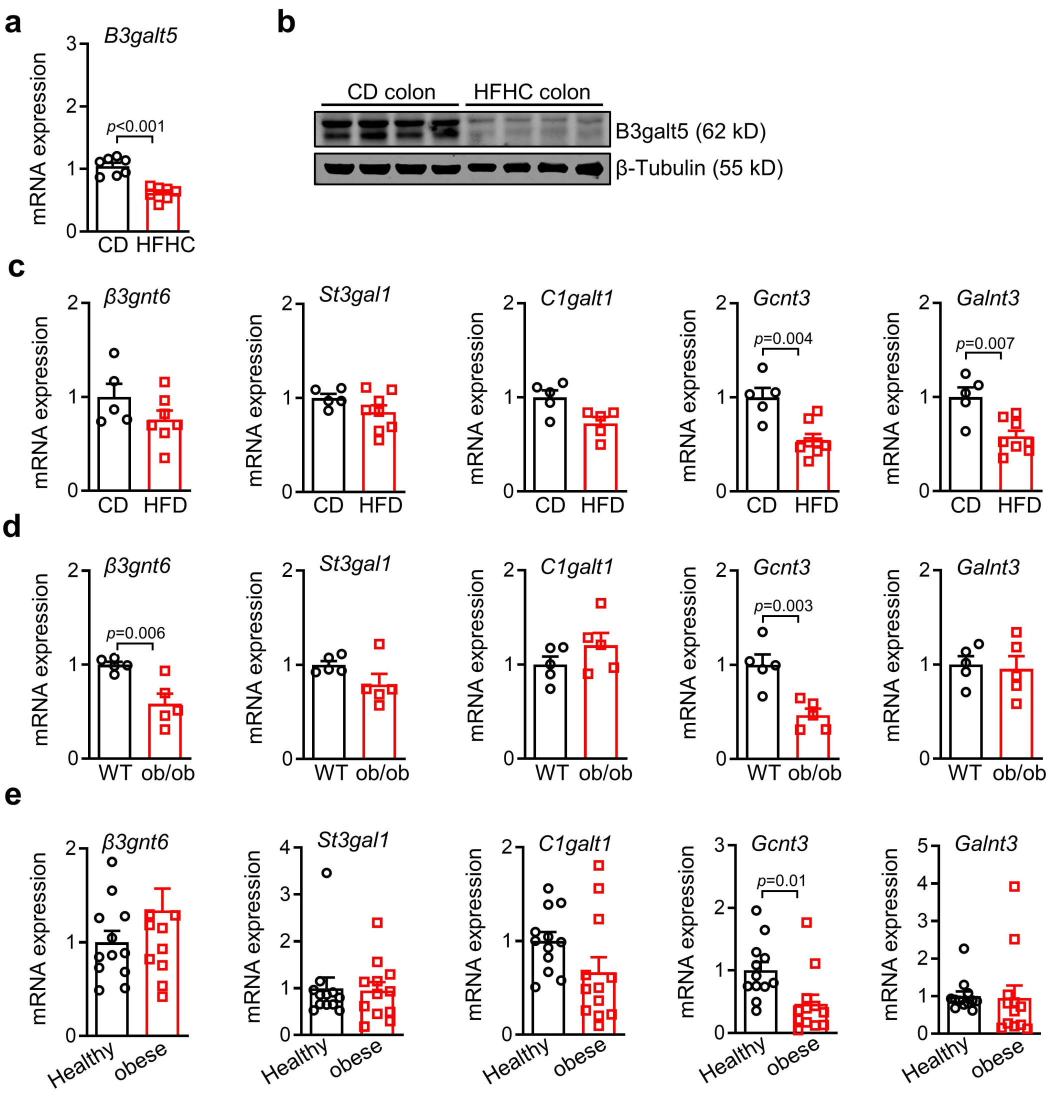

Figure S3. The expression of *O*-glycosyltransferase genes in intestine during obese. (a-b) The mRNA (a; *n* = 7 per group) and protein (b) expression of colonic B3galt5 in mice fed with 16-week CD or high fructose- and cholesterol (HFHC) diet. (c) Intestinal mRNA levels of *O*-glycosyltransferase genes in wild-type mice fed with chow diet or high-fat diet for 12 weeks (*n* = 5 for CD, *n* = 7 for HFD). (d) Intestinal mRNA levels of *O*-glycosyltransferase genes in wild-type mice (WT) and ob/ob mice (*n* = 5 per group). (e) Intestinal mRNA levels of *O*-glycosyltransferase genes in colon collected from healthy individuals and obese patients (*n* = 12 per group). Data are mean ± SEM. Significance was determined using unpaired two-tailed Student's t test. At least three independent experiments were repeated with similar results. Source data

are provided as a Source Data file.

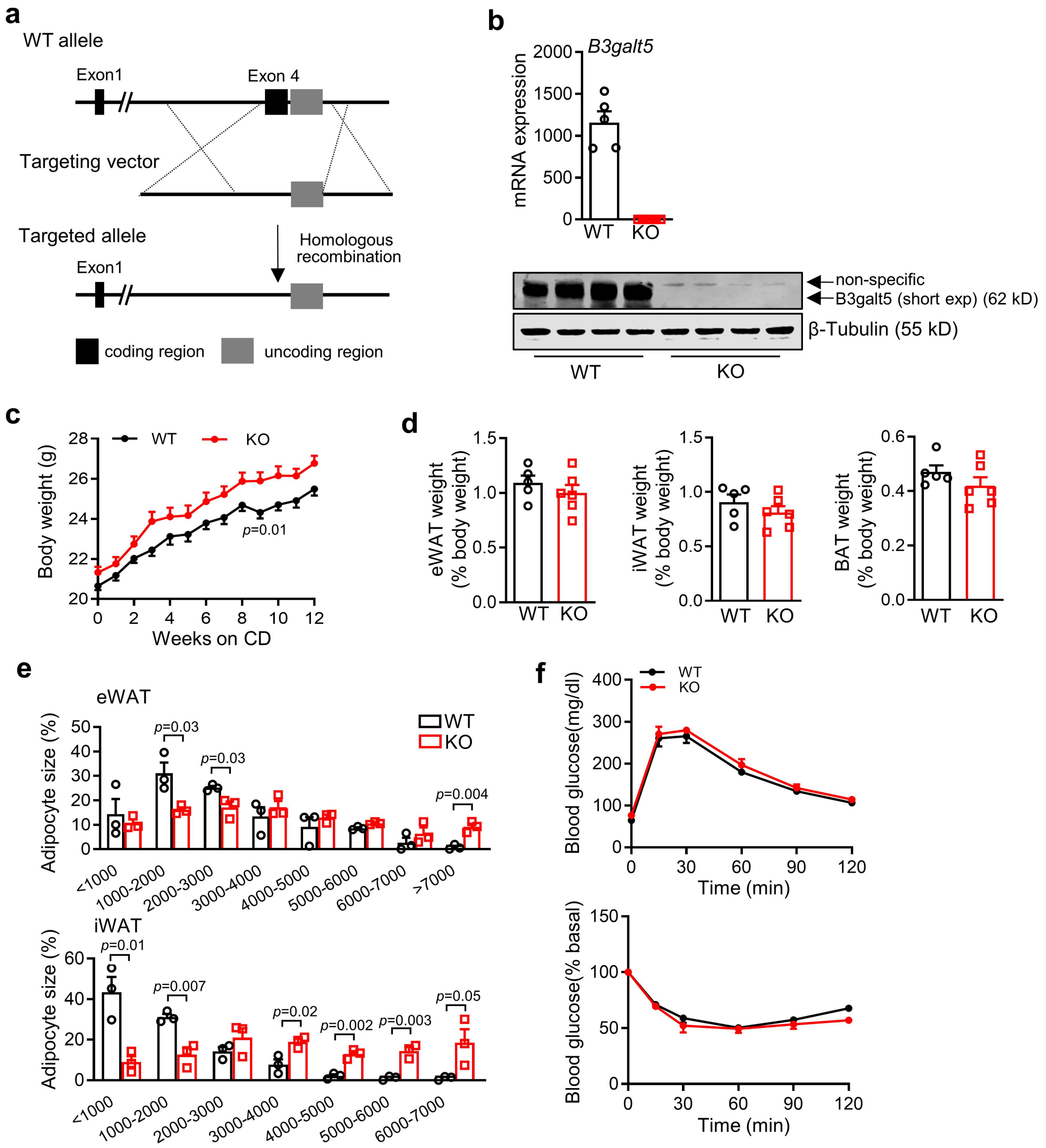

Figure S4. B3galt5 whole-body knockout has no influence on obesity and insulin resistance under chow diet. (a) Schematic showing the strategy for generating B3galt5 whole body knockout mice by homologous recombination. (b) The mRNA and protein levels of B3galt5 in intestine of WT and B3galt5 KO mice (n = 5 for WT, n = 6 for KO). (c) Growth curve of WT and B3galt5 KO mice fed with chow diet (CD) for 12 weeks (n = 12 per group). Significance was analyzed using two-way analysis of variance (ANOVA) with Sidak's multiple comparisons test. (d) The ratio of fat depots

to body weight in WT and B3galt5 KO mice fed with chow diet for 12 weeks (*n* = 5 for WT, *n* = 6 for KO). (e) Distribution of adipocyte size of eWAT and iWAT under HFD for 12 weeks (*n* = 3 per group). The statistical differences between groups were determined using one-way ANOVA with post-hoc Tukey test. (f) Blood glucose concentrations during GTT (2 g/kg) and ITT (0.5 U/kg) in WT and B3galt5 KO mice fed with chow diet for 12 weeks (*n* = 5 for WT, *n* = 6 for KO). WT: wild type; KO: B3galt5 whole-body knockout mice; eWAT: epididymal white adipose tissue; iWAT: inguinal white adipose tissue; BAT: brown adipose tissue; RER: respiratory exchange ratio. Data are mean ± SEM. At least three independent experiments were repeated with similar results. Source data are provided as a Source Data file.

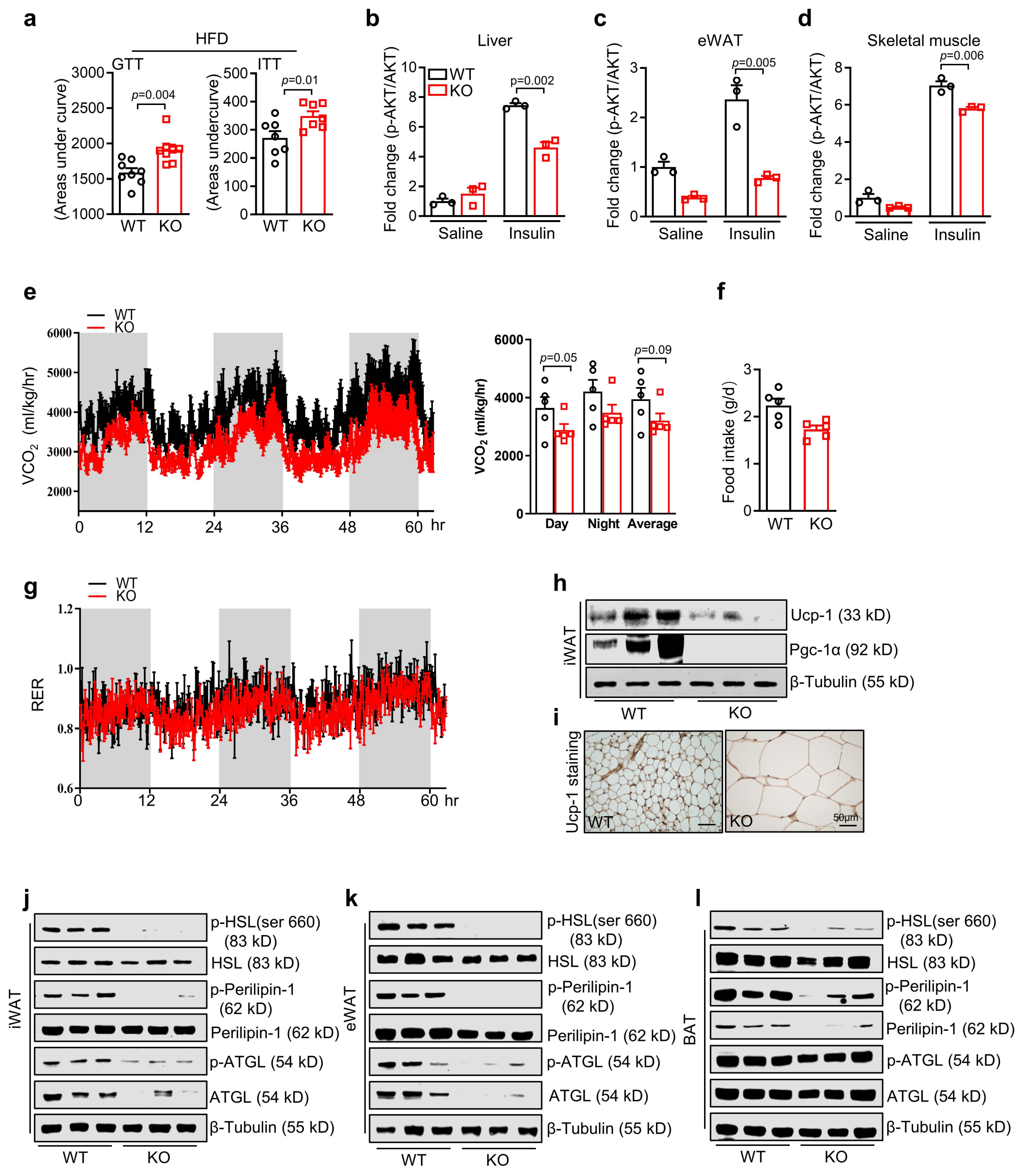

Figure S5. B3galt5 deficiency promotes high-fat diet induced insulin resistance and metabolic dysfunction. WT and B3galt5 KO mice were fed with HFD for 12 weeks. (a) The areas under curve (AUC) of GTT and ITT in WT and B3galt5 KO mice (*n* = 8 per group). (b-d) Semi-quantification analysis of p-Akt/Akt of WT and B3galt5 KO mice in liver (b), eWAT (c), Skeletal muscle (d) (*n* = 3 per group). (e) carbon dioxide consumption of WT and B3galt5 KO mice (*n* = 5 per group). (f) The food intake of WT and B3galt5 KO mice (*n* = 5 per group). (g) Respiration exchange

rate (RER) of WT and B3galt5 KO mice (*n* = 5 per group). (h) The protein levels of Ucp-1 and Pgc-1α in iWAT of WT and B3galt5 KO mice. (i) Ucp-1 IHC staining of iWAT. Scale bar: 50 μm. (j-l) Protein level of key lipolytic proteins in iWAT(j), eWAT(k), and BAT(l). HFD: high fat diet; GTT: glucose tolerance test; ITT: insulin tolerance test; eWAT: epididymal white adipose tissue; iWAT: inguinal white adipose tissue; BAT: brown adipose tissue; RER: respiratory exchange ratio; WT: wild type; KO: B3galt5 whole-body knockout mice. Data are mean ± SEM. The data sets (SFig. 5b-d) were analyzed using non-parametric approaches and the statistical differences between groups were determined using one-way ANOVA with post-hoc Tukey test. The remaining statistical differences were determined using unpaired two-tailed Student’s t test. At least three independent experiments were repeated with similar results. Source data are provided as a Source Data file.

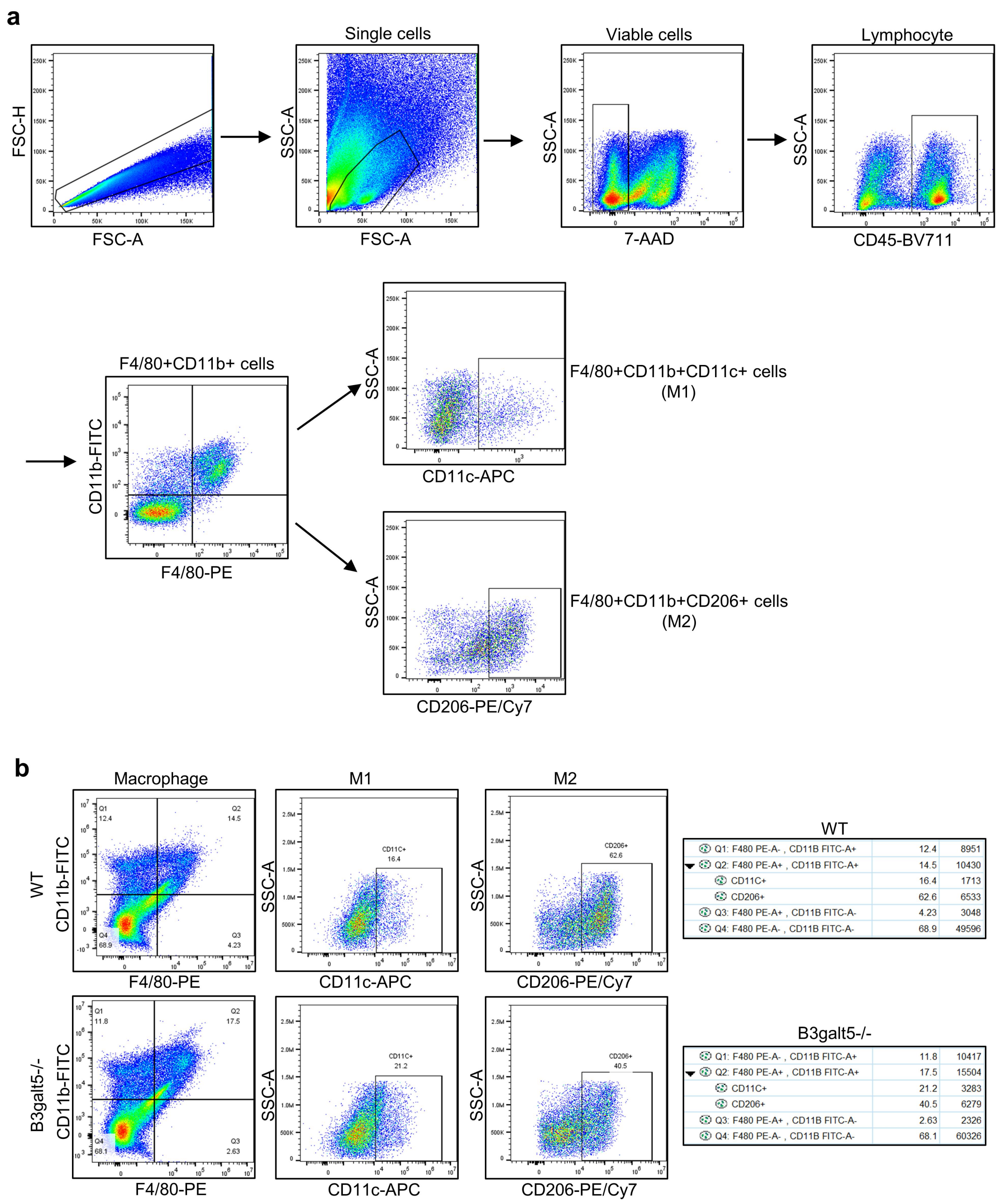

Figure S6. B3galt5 deficiency elevated the M1/M2 ratio of macrophages in adipose tissue. (a) Flow cytometry gating strategies for white adipose tissue. Different populations were identified based on a combination of marker expression a fluorescence minus one control. The isotype control for CD45, F4/80, CD11b, CD11c, and CD206 was utilized in the study. (b) FACS analysis of macrophages in eWAT of WT and B3galt5-/- mice fed an HFD for 12 weeks. WT: wild type; B3galt5-/-: B3galt5 whole-body knockout mice. Source data are provided as a Source Data file.

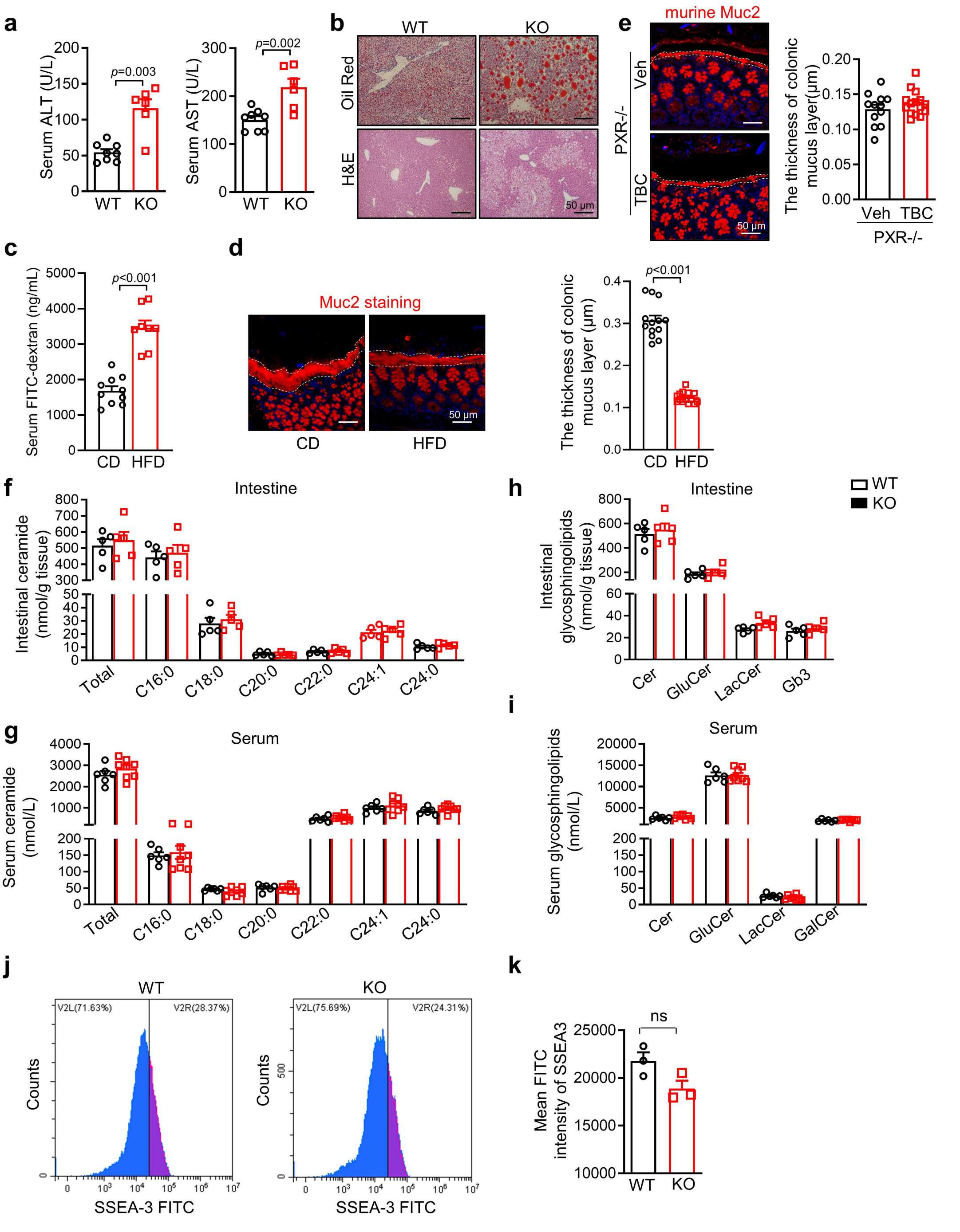

Figure S7. B3galt5 deficiency impaired hepatic steatosis, but has no influence on ceramides, GSLs and SSEA3 levels. (a) Serum ALT and AST level in WT and B3galt5 KO mice fed with high fat diet for 12 weeks (n = 8 for WT, n = 6 for KO). (b) H&E and Oil red O staining of liver tissue in WT and B3galt5 KO mice fed with high fat diet for 12 weeks. Scale bar: 50 μm. (c) The concentration of FITC-dextran in

serum of WT mice fed with CD or HFD for 12 weeks (*n* = 10 for CD, *n* = 8 for HFD). (d) Immunofluorescence staining of Muc2 of colon in WT mice fed with 12-week HFD (*n* = 13 for CD, *n* = 14 for HFD). Scale bar: 50 μm. (e) Muc2 staining of colon from PXR-/- mice treated with TBC. Scale bar: 50 μm. Quantification of mucus layer thickness (right; *n* = 12 for PXR-/- + Veh, *n* = 15 for PXR-/- + TBC). (f-g) Metabolomics analysis of ceramide content in intestine (f; *n* = 5 per group) and serum (g; *n* = 6 for WT, *n* = 8 for KO) of WT and B3galt5 KO mice. (h-i) Metabolomics analysis of glycosphingolipids content in intestine (h; *n* = 5 per group) and serum (i; *n* = 6–8 as (g)) of WT and B3galt5 KO mice. (j-k) Flow cytometry analysis of mean FITC intensity of SSEA3 in intestine of WT and B3galt5 KO mice (*n* = 3 per group). ALT: Alanine transaminase; AST: Aspartate transaminase; WT: wild type; KO: B3galt5 whole-body knockout mice; PXR-/-: PXR whole-body knockout mice; GSLs: glycosphingolipids; SSEA-3: stage-specific embryonic antigen 3. Data are mean ± SEM. The statistical differences were determined using unpaired two-tailed Student's t test. At least three independent experiments were repeated with similar results. Source data are provided as a Source Data file.

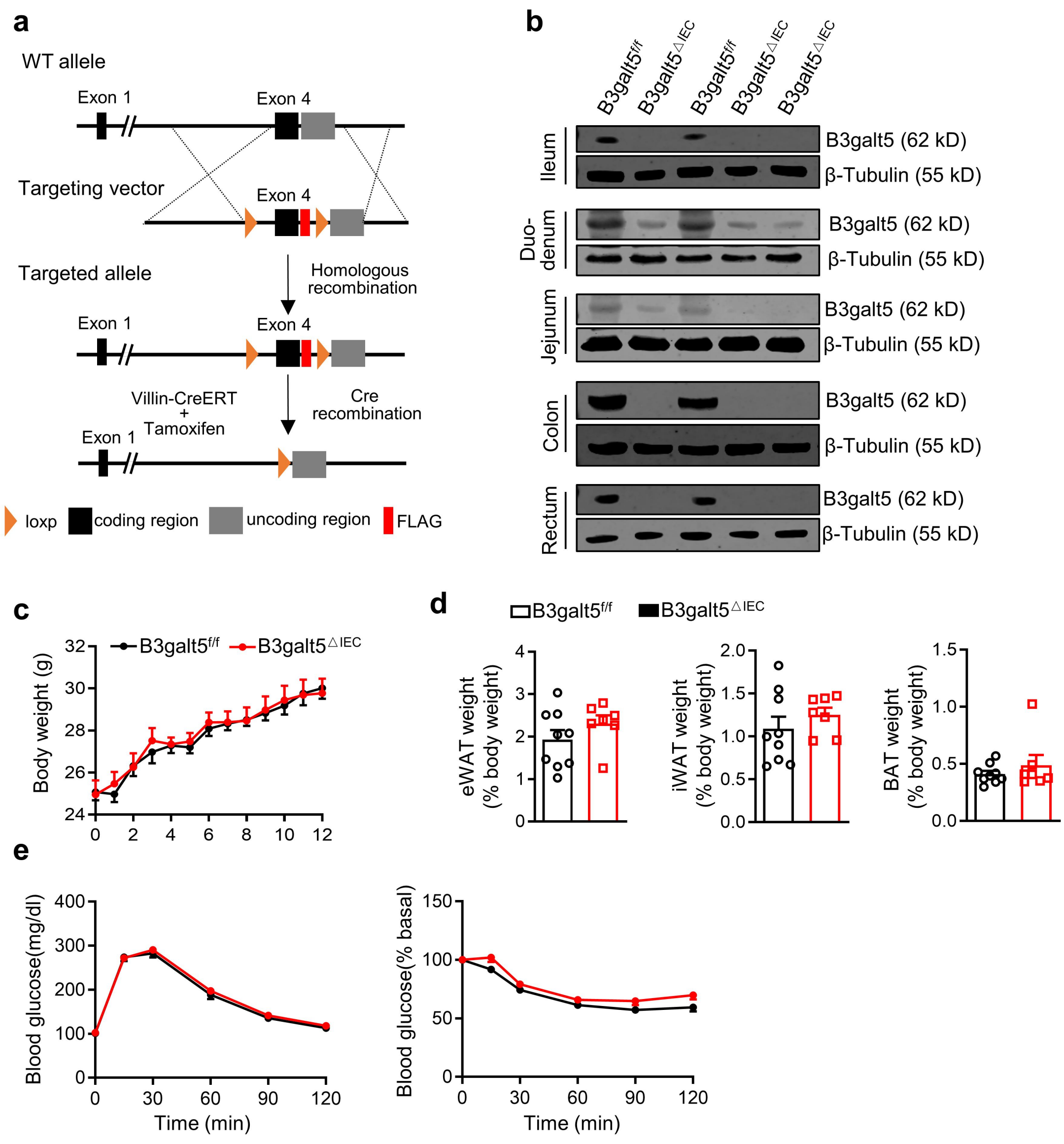

Figure S8. Intestinal specific B3galt5 knockout has no influence on obesity and insulin resistance under chow diet. (a) Schematic showing the strategy for generating intestinal specific B3galt5 knockout mice by homologous recombination (left). (b) Protein levels of B3galt5 in intestine of *B3galt5*$^{f/f}$ and *B3galt5*$^{\triangle IEC}$ mice. (c) Growth curve of *B3galt5*$^{f/f}$ and *B3galt5*$^{\triangle IEC}$ mice fed with chow diet for 12 weeks (n = 9 for *B3galt5*$^{f/f}$, n = 7 for *B3galt5*$^{\triangle IEC}$). (d) The ratio of fat depots to body weight (n = 7–9 as (c)). (e) Blood glucose concentrations during GTT (1 g/kg) and ITT (1.5 U/kg) in *B3galt5*$^{f/f}$ and *B3galt5*$^{\triangle IEC}$ mice fed with chow diet for 12 weeks (n = 7–9 as (c)). *B3galt5*$^{f/f}$: B3galt5 floxed mice; *B3galt5*$^{\triangle IEC}$: intestine-specific B3galt5-deficient mice;

eWAT: epididymal white adipose tissue; iWAT: inguinal white adipose tissue; BAT: brown adipose tissue. Data are mean ± SEM. At least three independent experiments were repeated with similar results. Source data are provided as a Source Data file.

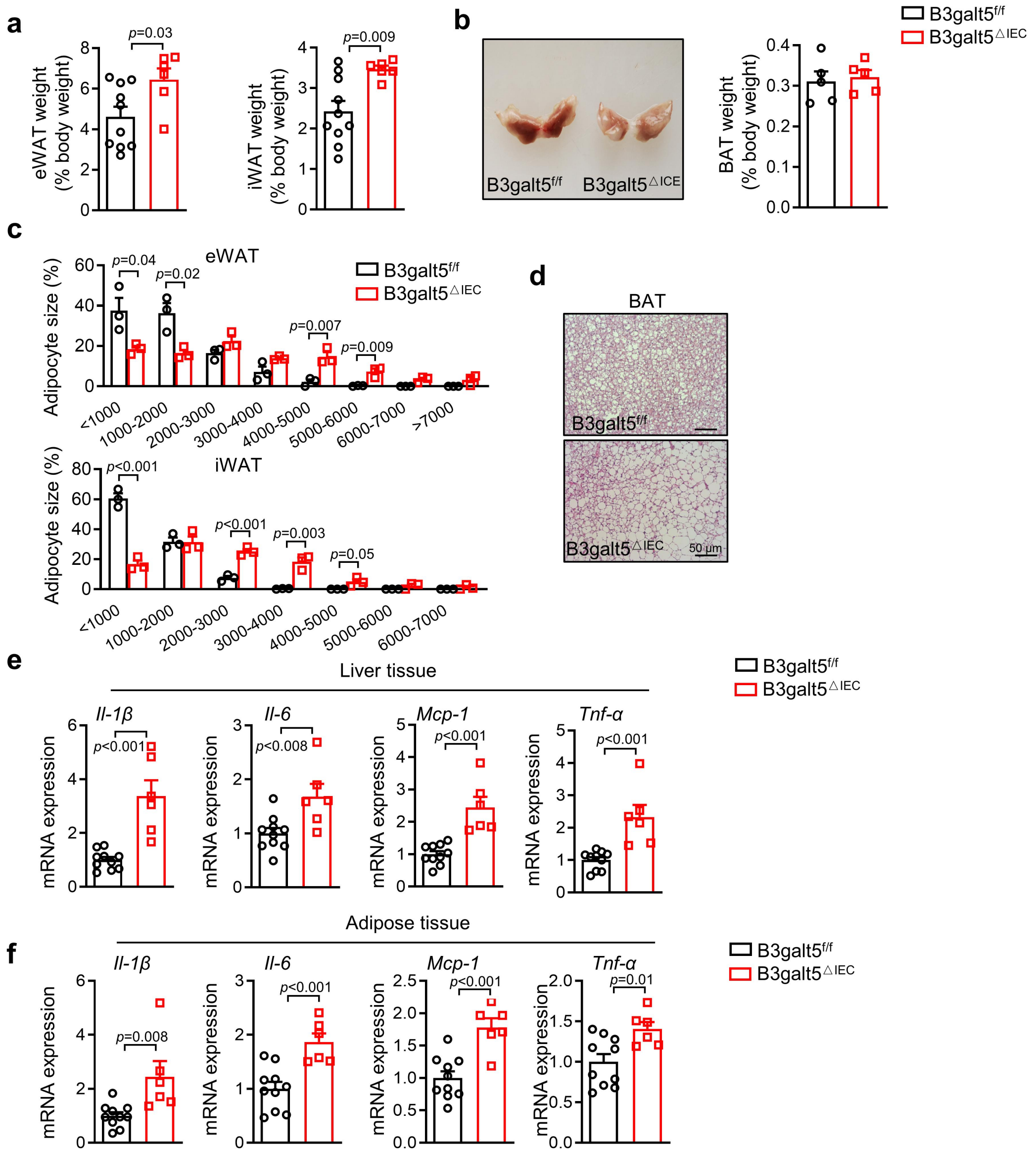

Figure S9. Intestinal specific B3galt5 knockout accelerates high-fat diet induced obesity. *B3galt5*$^{f/f}$ and *B3galt5*$^{\triangle IEC}$ mice were fed with HFD for 12 weeks. (a) The ratio of eWAT and iWAT depots to body weight (n = 10 for *B3galt5*$^{f/f}$, n = 6 for *B3galt5*$^{\triangle IEC}$). (b) Representative photographs of BAT (left) and the ratio of fat depots to body weight (right; n = 5 per group). (c) Distribution of adipocyte size of eWAT

and iWAT in *B3galt5*$^{f/f}$ and *B3galt5*$^{\triangle IEC}$ mice (*n* = 3 per group). The statistical differences between groups were determined using one-way ANOVA with post-hoc Tukey test. (d) H&E staining of BAT in *B3galt5*$^{f/f}$ and *B3galt5*$^{\triangle IEC}$ mice. Scale bar: 50 μm. (e) The mRNA expression of inflammation related gene *Il-1β, Il-6*, *Mcp-1* and *Tnf-α* expression in liver (*n* = 6–10 as (a)). (f) The mRNA expression of inflammation related gene *Il-1β, Il-6*, *Mcp-1* and *Tnf-α*, expression in adipose tissue (*n* = 6–10 as (a)). *B3galt5*$^{f/f}$: B3galt5 floxed mice; *B3galt5*$^{\triangle IEC}$: intestine-specific B3galt5-deficient mice; eWAT: epididymal white adipose tissue; iWAT: inguinal white adipose tissue; BAT: brown adipose tissue. Data are mean ± SEM. The remaining statistical differences were determined using unpaired two-tailed Student's t test. At least three independent experiments were repeated with similar results. Source data are provided as a Source Data file.

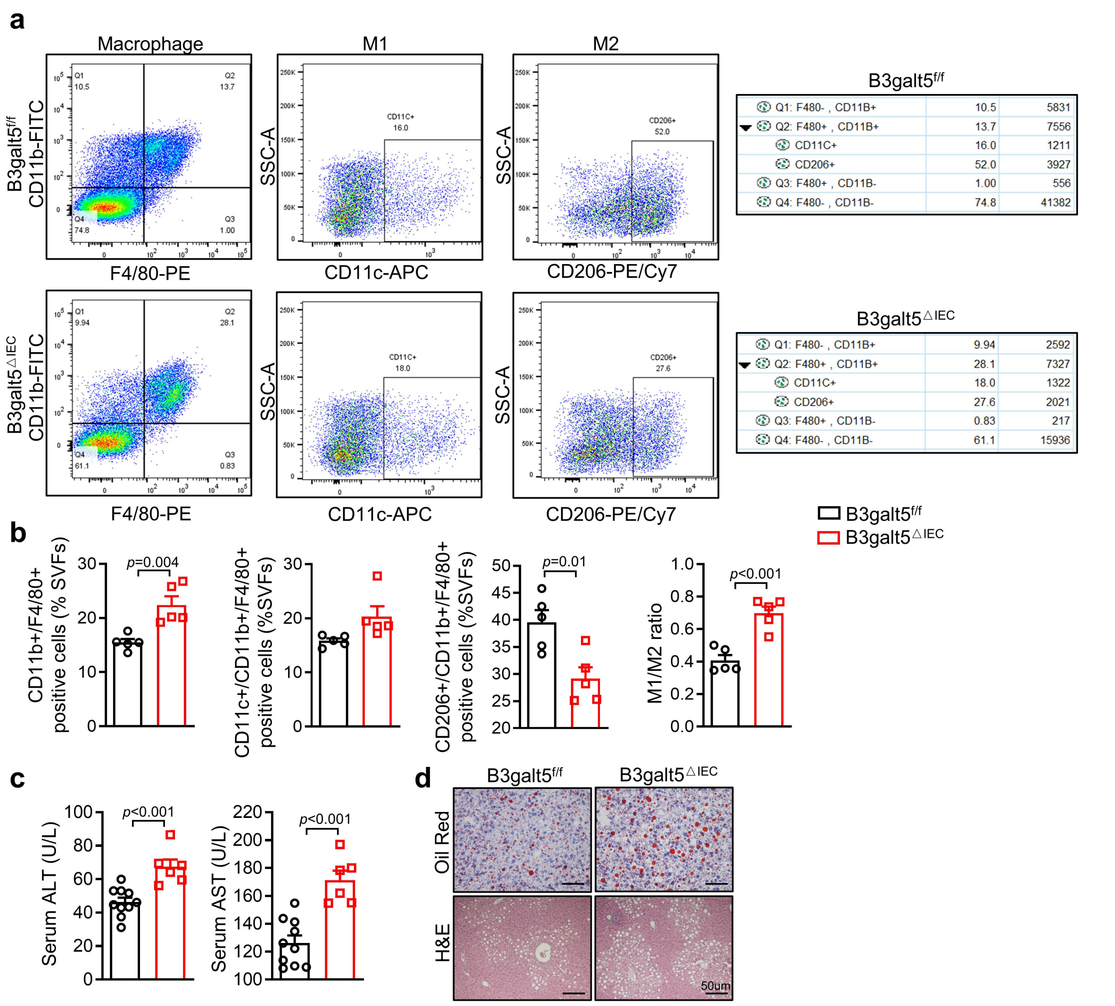

Figure S10. Intestinal specific B3galt5 knockout accelerates high-fat diet induced inflammation in adipose tissue and hepatic steatosis. *B3galt5*$^{f/f}$ and *B3galt5*$^{\triangle IEC}$ mice were fed with HFD for 12 weeks. (a) FACS analysis of macrophages in eWAT of *B3galt5*$^{f/f}$ and *B3galt5*$^{\triangle IEC}$ mice. (b) Percentage of macrophages, M1 and M2 macrophages, and ratio of M1 to M2 (n = 5 per group). (c)Serum ALT and AST level in *B3galt5*$^{f/f}$ and *B3galt5*$^{\triangle IEC}$ mice fed with high fat diet for 12 weeks (n = 10 for *B3galt5*$^{f/f}$, n = 6 for *B3galt5*$^{\triangle IEC}$). (d) H&E and Oil red O staining of liver tissue in *B3galt5*$^{f/f}$ and *B3galt5*$^{\triangle IEC}$ mice fed with high fat diet for 12 weeks. Scale bar: 50 μm. *B3galt5*$^{f/f}$: B3galt5 floxed mice; *B3galt5*$^{\triangle IEC}$: intestine-specific B3galt5-deficient mice; ALT: Alanine transaminase; AST: Aspartate transaminase. Data are mean ± SEM. The statistical differences were determined using unpaired two-tailed Student's t test. At

least three independent experiments were repeated with similar results. Source data are provided as a Source Data file.

Supplementary Tables

Table S1. Structural characterization of reduced O-linked oligosaccharides from colonic Muc2 from WT and *B3galt5-/-* mice.

Code	Glycan	m/z	Retention time(min)	Relative abundance(%): Mean±SEM	
Neutral				WT	KO
384.15	Gal-GalNAcol	384.15	2.82	5.72±0.74	3.92±0.71
425.18	HexNAc-GalNAcol	425.18	3.04	0.22±0.09	1.72±0.11
425.18	HexNAc-GalNAcol	425.18	5.48	1.08±0.46	0.14±0.10
530.21	Fuc-Gal-GalNAcol	530.21	15.28	21.61±3.22	32.37±3.94
587.23	Gal-(HexNAc-6)GalNAcol	587.23	6.62	20.95±2.00	11.03±1.28
733.29a	Fuc-Gal-GlcNAc-GalNAcol	733.29	10.41	0.12±0.04	ND
733.29b	Fuc-Gal-(HexNAc-6)GalNAcol	733.29a	15.95	6.54±0.48	15.10±0.99
749.28	Gal-(Gal-4GlcNAc-6)GalNAcol	749.28	9.99	0.41±0.07	0.28±0.07
790.31	HexNAc-Gal-(HexNAc-6)GalNAcol	790.31	9.12	0.18±0.02	0.78±0.08
895.34a	Gal-(Fuc-Gal-GlcNAc-6)GalNAcol	895.34	11.3	0.70±0.18	0.11±0.02
895.34b	Gal-(Fuc-Gal-4GlcNAc-6)GalNAcol	895.34	13.43	1.18±0.35	0.73±0.19
895.34c	Fuc-Gal-(Gal-GlcNAc-6)GalNAcol	895.34	16.67	1.63±0.16	1.63±0.23
1041.4a	Fuc-Gal-(Fuc-Gal-GlcNAc-6)GalNAcol	1041.4	17.51	1.27±0.25	ND
1041.4b	Fuc-Gal-(Fuc-Gal-4GlcNAc-6)GalNAcol	1041.4	18.92	6.05±1.98	17.65±3.82
1098.42	HexNAc-Gal-(Fuc-Gal-4GlcNAc-6)GalNAcol	1098.42	15.22	0.18±0.04	0.4±0.12
1244.48a	Fuc-Gal-GlcNAc-(Fuc-Gal-GlcNAc-6)GalNAcol	1244.48	17.22	3.08±1.95	ND
1244.48b	Fuc-Gal-GlcNAc-(Fuc-Gal-4GlcNAc-)GalNAcol	1244.48	18.27	0.70±0.11	1.56±0.29
One acidic residue					
667.19	Gal-(SO_3^--GlcNAc-6)GalNAcol	667.19	17.06	3.28±0.36	1.99±0.30
675.25	[NeuAc]$_1$Hex-GalNAcol	675.25	7.37	0.31±0.13	0.29±0.02
829.24	Gal-(Gal-(SO_3^--)GlcNAc-)GalNAcol	829.24	15.94	0.29±0.07	0.05±0.01
829.24	Gal-(SO_3^--Gal-GlcNAc-)GalNAcol	829.24	20.69	0.36±0.06	0.19±0.04
878.33a	Gal-(NeuAc-)GlcNAc-GalNAcol	878.33	17.82	0.09±0.03	ND
878.33b	NeuAc-GlcNAc-Gal-GalNAcol	878.33	19.2	ND	0.25±0.01
975.3	Fuc-Gal-(SO_3^--Gal-GlcNAc-)GalNAcol	975.3	19.37	2.21±0.56	0.71±0.08
1040.38a	Gal-(Gal-(NeuAc-)GlcNAc-)GalNAcol	1040.38	16.77	0.27±0.12	0.54±0.11
1040.38b	Gal, Gal-(NeuAc-)GlcNAc-GalNAcol	1040.38	19.9	0.62±0.13	ND
1081.41	[NeuAc]$_1$HexNAc-Gal-GlcNAc-GalNAcol	1081.41	21.56	0.47±0.24	ND
1121.36	Fuc-Gal-3(Fuc-Gal-4(SO_3^--)GlcNAc-6)GalNAcol	1121.36	18.39	0.97±0. 42	1.16±0.95
1186.44	Fuc-Gal-(Gal-(NeuAc-)GlcNAc-)GalNAcol	1186.44	24.42	1.27±0. 23	0.27±0. 05
1243.46a	[NeuAc]$_1$Gal-(HexNAc-Gal-4GlcNAc-6)GalNAcol	1243.46	18.26	2.04±0. 21	3.00±0. 54
1243.46b	[NeuAc]$_1$Gal-(GlcNAc-Gal-GlcNAc-)GalNAcol	1243.46	19.13	0.99±0. 12	1.08±0. 08
1389.52a	Fuc-Gal-(NeuAc,HexNAc-Gal-4GlcNAc-6)GalNAcol	1389.52	17.68	0.12±0. 12	1.05±0. 28
1389.52b	Gal-(NeuAc-GlcNAc(Fuc-Gal-GlcNAc-6)GalNAcol	1389.52	24.49	13.77±5.47	ND
Multiple acidic residues					

1120.34a	Gal-(SO_3^--Gal-(NeuAc-)GlcNAc-)GalNAcol	1120.34	13.93	ND	0.07±0. 02
1120.34b	SO_3^--Gal-(NeuAc-)GlcNAc-Gal-GalNAcol	1120.34	18.94	ND	0.09±0. 03
1266.39	[NeuAc]$_1$Fuc-Gal-(Gal-(SO_3^--)GlcNAc-)GalNAcol	1266.39	18.17	ND	0.11±0. 02
1331.47a	NeuAc-Gal-(Gal-(NeuAc-)GlcNAc-)GalNAcol	1331.47	17.79	ND	0.11±0. 02
1331.47b	NeuAc-Gal-(NeuAc-)GlcNAc-Gal-GalNAcol	1331.47	19.8	ND	0.06±0. 01
1372.5	HexNAc-(NeuAc-)Gal-(NeuAc-)GlcNAc-GalNAcol	1372.5	18.65	0.08±0. 03	0.08±0. 07
1469.47	[NeuAc]$_1$Fuc-Gal-(HexNAc-Gal-(SO_3^--)GlcNAc-)GalNAcol	1469.47	19.17	0.06±0. 02	0.07±0. 01
1526.49	[SO_3^-]$_1$ [NeuAc]$_1$ [HexNAc]$_3$[Gal]$_2$GalNAcol	1526.49	15.96	0.04±0. 01	0.11±0. 02
1565.42	[NeuAc]$_1$Gal-(SO_3^--Gal-GlcNAc-Gal-(SO_3^--6)GlcNAc-)GalNAcol	1565.42	15.95	0.24±0. 03	0.40±0. 03
1680.61a	Fuc-Gal-(HexNAc-(NeuAc-)Gal-(NeuAc-)GlcNAc-)GalNAcol	1680.61	17.07	0.11±0. 08	0.04±0. 02
1680.61b	NeuAc-Gal-(NeuAc-)GlcNAc-(Fuc-Gal-4GlcNAc-6)GalNAcol	1680.61	18.54	0.13±0. 08	ND
1737.63	[NeuAc]$_2$GlcNAc-Gal-(HexNAc-Gal-GlcNAc-)GalNAcol	1737.63	25.29	0.08±0. 02	0.14±0. 03
1768.5	[NeuAc]$_1$Gal-(HexNAc-(SO_3^-)-Gal-GlcNAc-Gal-(SO_3^--6)GlcNAc-)GalNAcol	1768.5	13.5	0.03±0. 01	0.06±0. 01
1834.6	[SO_3^-]1 [NeuAc]$_1$[Fuc]$_1$[HexNAc]$_3$[Gal]$_3$GalNAcol	1834.6	18.93	0.02±0. 01	0.07±0. 02
1842.66a	[NeuAc]$_2$Gal, Gal-GlcNAc-(Fuc-Gal-4GlcNAc-6)GalNAcol	1842.66	17.08	0.10±0. 04	ND
1842.66b	[NeuAc]$_2$[Fuc]$_1$[HexNAc]$_2$[Gal]$_3$GalNAcol	1842.66	18.63	0.07±0. 04	0.12±0. 04
1883.69	[NeuAc]$_1$NeuAc,HexNAc-Gal-GlcNAc-(Fuc-Gal-4GlcNAc-6)GalNAcol	1883.69	11.89	0.08±0. 03	0.05±0. 02
1940.71a	[NeuAc]$_2$ HexNAc-Gal-GlcNAc-(HexNAc-Gal-GlcNAc-)GalNAcol	1940.71	17.07	0.07±0. 02	ND
1940.71b	[NeuAc]$_2$[HexNAc]$_4$[Gal]$_2$GalNAcol	1940.71	18.62	0.06±0. 03	0.09±0. 03
1979.64	[SO_3^-]$_1$[NeuAc]$_2$[HexNAc]$_3$[Gal]$_3$GalNAcol	1979.64	25.22	0.10±0. 02	0.23±0. 10
2028.73	[NeuAc]$_3$Gal-GlcNAc-(HexNAc-Gal-GlcNAc-)GalNAcol	2028.73	13.43	0.06±0. 02	0.07±0. 02
Core 1 and Core 2				79.57±4.53	98.05±0. 35
Core 3 and Core 4				20.43±4.53	1.95±0. 35

Table S2. Demographic and pathological characteristics of patients with obesity.

	Healthy (BMI<24 kg/m^2)	Overweight (BMI: 24.0-27.9 kg/m^2)	Obese (BMI⩾28.0 kg/m^2)
**Number of patients**	24	3	27
**Age (years)**	37.58±2.17	37.33±6.33	42.85±1.86
**Gender**			
Male	9	1	17
Female	15	2	10
**BMI**	20.32±0.32	25.33±1.04	29.07±0.21
**Etiology**	Healthy at the end of ileum and colorectal mucosa	Healthy at the end of ileum and colorectal mucosa	Healthy at the end of ileum and colorectal mucosa

Table S3. Real-time PCR primers.

Genes	sense (5'- 3')	anti-sense (5'- 3')
m18s	TTGACTCAACACGGGAAACC	AGACAAATCGCTCCACCAAC
mB3galt5	TGATGGGTATGGAATGGG	AGCTTGATGAACGGAACG
mCyp3a11	CTCAATGGTGTGTATATCCCC	CCGATGTTCTTAGACACTGCC
mB3galt1	CCACAGTGGCACATCTACCGTTG	TGGCTCACCTTGTCTTGCTTCATC
mB3galt2	AAGCCAGACCTGCCTCCTAGAC	TCGCCAGTGATTGAACACGAACTC
mB3galnt1	TGATGCTGAGGAACACCACATGC	AGCCTGAGCCATCCAGACTGTC
mB3galnt4	CCTGGCGGTCCTACTACTGGTG	AGGAGGAAGAGCGTCTGCACTC
mB3galnt6	CAGCCTGGTCTACAGAGTGAGTCC	CCAAGTGCCTTACAGTCCATACGC
mC1galt1	ATGGCCTCTAAATCTTGGCTGA	AGCCTCTTCTCGCAACAAAATA
mSt3galt1	TCCAACACGGGAGTACCCA	GCTGGTCGAACCAATATGATACC
mB3gnt6	AAGAGTCCCACGACACTGG	GTAGCGCAGGAAGTCTTGGA
mGcnt3	TAGCAAGCCGAGTAGAAG	CAACCAAACTAAGCTCCA
mGalnt3	TGCAAATAGGAGCGCCCATTA	GGCGATCAAAAACCGGCTTC
mMcp1	GCTGGAGAGCTACAAGAGGATC	GTCAACTTCACATTCAAAGTGC
mTnfa	GGCGGTGCCTATGTCTCA	AGGGTCTGGGCCATAGAA
mIl-1β	TGCCACCTTTTGACAGTGATG	CAAAGGTTTGGAAGCAGCC
mPai-1	TTCAGCCCTTGCTTGCCTC	ACACTTTTACTCCGAAGTCGGT
mF4/80	TGTCTGAAGATTCTCAAAACATGGA	GTGTTTCTCCTTGGTGCATA
mCd68	TCGCCTAGTCCAAGGTCCAA	GGTACCGTCACAACCTCCC
mCd11b	TTTTAGGAGCACCTCGGTAT	TGAGGATCAAGTTGGTATTG
mCd11c	AAAATCTCCAACCCATGCTG	CACCACCAGGGTCTTCAAGT
mIcam1	TGCCTCTGAAGCTCGGATATAC	TCTGTCGAACTCCTCAGTCAC
mGLP-1	TTACTTTGTGGCTGGATTGCTT	AGTGGCGTTTGTCTTCATTCA
mPYY	ACGGTCGCAATGCTGCTAAT	GACATCTCTTTTTCCATACCGCT
mCCK	CCCTGACATTGACACCTCCT	AGCCTTTCCTGGTGGAGATT
mB3galt5-Chip	CTGCCACCAGACTAAAGG	CATGGACAGGAGGTCACTA
hGAPDH	ACAACTTTGGTATCGTGGAAGG	GCCATCACGCCACAGTTTC
h18s	ACACGGACAGGATTGACAGA	GGACATCTAAGGGCATCACAG
hMDR1	AAAAAGATCAACTCGTAGGAGTA	GCACAAAATACACCAACAA
hB3galt5	TCCTCTTGGCATTTACACT	AGTTCCCGTCTTTCTTGTA
hB3gnt6	GTGCGCCGCCTCTTTCTATT	CCAGCCAGTCGAGCAAGTG
hSt3galt1	AAGAGGACCCTGAAAGTGCTC	CTCCAGGACCATCTGCTTGG
hC1galt1	TCCTCTGTGGATCAGCAATAGG	TTAGGCTGGGTGTCAACCTTT
hGcnt3	TCAAAGAGGCGGTCAAAGCAA	GCATAAACCACCCGAACCAG
hGalnt3	CAGCAGAATTGAAGCCTGTCC	CTTCCCCACGTTCCTTTTCCT

Table S4. Antibodies used

Antibody	Catalog	Company	Application	Dilution
B3galt5	E9390	ABclonal Technology co.ltd, China	WB/IF	1:1000/1:200
B3galt5	SAB1302633	Sigma-Alrich, St. Louis, MO, USA	WB	1:1000
p-Akt (Ser473)	Sc-7985	Santa Cruz Biotechnology, Santa Cruz, CA	WB	1:1000
Akt	BM4400	Boster Biological Technology co.ltd, China	WB	1:1000
Muc2	30EE6A15	Invitrogen, Thermo Fisher Scientific, CA	WB/IF	1:1000/1:200
PXR	sc-48340	Santa Cruz Biotechnology, Santa Cruz, CA	ChIP	1:200
β-catenin	sc-7963	Santa Cruz Biotechnology, Santa Cruz, CA	IF	1:200
Ucp-1	Ab10983	Abcam, Cambridge, MA	WB	1:1000
Pgc-1α	sc-517380	Santa Cruz Biotechnology, Santa Cruz, CA	WB	1:1000
p-HSL	4126	Cell Signaling Technology, USA,	WB	1:1000
HSL	sc-25843	Santa Cruz Biotechnology, Santa Cruz, CA	WB	1:1000
p-Perilipin	100G7E	Cell Signaling Technology, USA	WB	1:1000
Perilipin	9349	Cell Signaling Technology, USA	WB	1:1000
p-ATGL	Ab135093	Abcam, Cambridge, MA	WB	1:1000
ATGL	2138s	Cell SignalingTechnology, USA	WB	1:1000
CD16/32	553141	BD Biosciences, Franklin, NJ	FC	1:500
CD45-BV711	407-0451-82	eBioscience, San Diego, CA	FC	1:200
Cd11b-FITC	561688	BD Biosciences, Franklin, NJ	FC	1:200
Cd206-PE/Cy7	141719	BioLegend, CA	FC	1:200
Cd11c-APC	561119	BD Biosciences, Franklin, NJ	FC	1:200
7-AminoactinoMycin D	559925	BD Biosciences, Franklin, NJ	FC	1:200
Rat IgG2b kappa Isotype Control (eB149/10H5)	407-4031-81	eBioscience, San Diego, CA	FC	1:200
FITC Rat IgG2b, κ Isotype Control	553988	BD Biosciences, Franklin, NJ	FC	1:200
PE/Cyanine7 Rat IgG2a, κ Isotype Ctrl	400521	BioLegend, CA	FC	1:200
APC Hamster IgG1, λ1 Isotype Control	553956	BD Biosciences, Franklin, NJ	FC	1:200
Rat IgG2a kappa Isotype Control (eBR2a), PE	12-4321-80	eBioscience, San Diego, CA	FC	1:200
SSEA-3-FITC	560236	BD Biosciences, Franklin, NJ	FC	1:200
F4/80	ab6640	Abcam, Cambridge, MA	IF	1:200
F4/80-PE	12-4801-80	eBioscience, San Diego, CA	FC	1:200
β-tubulin	200608	Zen Bio Science, China	WB	1:4000
Goat anti-Rabbit	966-32210	Licor	WB	1:20000
Goat anti-Mouse	966-32211	Licor	WB	1:20000

Supplementary Methods

Animal treatment

To induce the recombination, mice were injected with tamoxifen (100 mg/kg) every other day for five times. For obesity model, 8-week-old male mice were fed with high fat diet (60%kJ fat, research diet, D12492) for 12 weeks. For specific activation of intestinal PXR, tributyl citrate (TBC; J&K scientific company, cat 179568) was mixed with powdered high-fat diet at 0.05% (w/w). 8-week-old male mice were fed with high-fat diet with TBC or vehicle for 12 weeks. For PXR activation model, 8-week-old male mice were injected with 40 mg/kg prenenolone-16a-carbonitrile (PCN; in DMSO/corn oil 1:3, Cayman Chemical Company, cat 16343) intraperitoneally every 8 h for three times. For fructose-induced metabolic disease model, 8-week-old male mice were fed a 40% fat and 0.2% cholesterol diet and given access to fructose-supplemented water (23.9g/L).

Cell culture

The human colon cancer cell line LS174T (ATCC, Cat#CL-188, isolated from the colon of a White, 58-year-old, female adenocarcinoma patient with colorectal cancer) and human embryonic kidney 293 (HEK293; ATCC, Cat#CRL-1573, isolated from the kidney of a human embryo) cells were cultured in Dulbecco's modified Eagle's medium (DMEM; high glucose, 4.5 g/L) supplemented with 10% (v/v) fetal bovine serum (FBS; Hyclone, USA), 50 ug/mL streptomycin, and 50 U/mL penicillin, and incubated at 37°C with 5% $CO2$.

RNA sequence

8-week-old mice were injected with 40 mg/kg prenenolone-16a-carbonitrile (PCN; in

DMSO/corn oil 1:3, Cayman Chemical Company, cat 16343) intraperitoneally every 8 h for three times. Mice were sacrificed 8 h after the last administration and the colon tissues were collected for total RNA isolation according to the manufacture's instruction. For the RNA-seq, libraries were constructed using 400 ng of total RNA and TruSeq RNA Sample Prep (Illumina) with Poly-A pull down rRNA depletion following manufacturer's recommendations. Samples were sequenced on the HiSeq2500 platform, using a read length of 100 bp paired-end reads. Before alignment, reads with a low quality and adapters were detected using FastQC and removed. Remaining reads were mapped to the SScrofa10.2.72 genome using default parameters in STAR aligner. This resulted in an average of 30,557,234 uniquely mapped reads per sample, of which on average 81.60 percent was mapped in the intragenic region (within introns or exons). On average 20,390 transcripts were detected among the mapped reads. Read counts were estimated at gene-level using HTSeq.

Flow cytometry

For macrophage infiltration analysis, stromal vascular fraction (SVF) from epidydimal white adipose tissue (eWAT) was isolated with collagenase I (Signa-Aldrich) for 20 min at 37°C. The suspension was filtered with 150 mesh filter and then centrifuged at 500g, for 5 min at 4°C to remove adipocyte from SVF. The SVF was then incubated with red blood cell lysis buffer (Beyotime, C3702) for 5 min at room temperature. SVF was resuspended in PBS containing 1mM EDTA, 25 mM HEPES, and 1%FBS after centrifugation. After incubating with Fc-block anti-CD16/32 for 10 min at 4°C, SVF was then stained with fluorescence-labeled primary antibodies for 30min on ice. Before analysis, SVF was incubated with 7-

AminoactinoMycin D for 10 min at room temperature to exclude the apoptosis cells. After excluding non-specific staining and adjusting fluorescence compensation by staining cells with single fluorescence-labeled primary antibodies, the SVF stained with mix antibodies was analyzed by CytoFlex (BD FACSAria™ Fusion, USA). The relative isotype control for CD45, F4/80, CD11b, CD11c and CD206 was utilized to identify positive cells. The gating strategy is shown in Fig S6A. For SSEA3 expression analysis, cells from the intestine from WT and B3galt5-/- mice were isolated and stained fluorescence-labeled primary antibodies and analyzed by CytoFlex (Beckman, CA). All antibodies used are in Supporting Table S4.

Histological analysis and immunostaining

For histological analysis, liver or adipose tissues were fixed with 10% formalin, embedded in paraffin, sectioned at 4 μm and stained hematoxylin and eosin (H&E). Segments of the distal colon without washing from mice were fixed in Methanol-Carnoy's fixative (60% methanol, 30% chloroform and 10% acetic acid) for 36 h. Paraffin-embedded sections were dewaxed and hydrated. Sections were stained with Alcian blue. Images were captured under a microscope (Nikon, Tokyo).

For immunofluorescence staining, tissue sections were fixed with 4% paraformaldehyde for 8 minutes, then permeabilized with 0.1M phosphate-buffered saline containing 0.2% Triton X-100 for 15 minutes. Tissue samples were incubated with primary antibodies at 4°C overnight. All antibodies used are in Supporting Table S4.

Tissue inflammatory cytokines analysis

To analyze hepatic and adipose inflammatory cytokines, 50 mg liver tissue and

adipose tissue was homogenized in 0.5 mL PBS. The supernatants from tissue homogenates were measured by using Elisa Kit from Invitrogen (88-7013-22, 88-7064-88, 88-7391-22, 88-7324-22).

Serum endotoxin detection

Serum endotoxin level from portal vein was detected by using Chromogenic LAL Endotoxin Assay Kit (Beyotime, C0276S).

Hepatic triglyceride content analysis

Hepatic lipid was extracted from 50 mg liver tissue via chloroform/methanol (2:1, vol/vol) and dissolved in 1% Triton X-100 in ethanol. Hepatic triglycerides content was analyzed via using commercial kits (Biosino, Beijing, China) and normalized to liver weight.
