# Supplementary material for: B3galt5 functions as a PXR target gene and regulates obesity and insulin resistance by maintaining intestinal integrity
Source: Nat Commun. 2024 Jul 14;15:5919. doi: 10.1038/s41467-024-50198-z (PMC11247088; doi:10.1038/s41467-024-50198-z)
Supplement: Supplementary file 3 — Reporting Summary [file 41467_2024_50198_MOESM3_ESM.pdf]

nature portfolio

Corresponding author(s): Jinhan He and Yanping Li

Last updated by author(s): Jul 1, 2024

Reporting Summary

Nature Portfolio wishes to improve the reproducibility of the work that we publish. This form provides structure for consistency and transparency in reporting. For further information on Nature Portfolio policies, see our Editorial Policies and the Editorial Policy Checklist.

Statistics

For all statistical analyses, confirm that the following items are present in the figure legend, table legend, main text, or Methods section.

n/a	Confirmed	
☐	☒	The exact sample size (n) for each experimental group/condition, given as a discrete number and unit of measurement
☐	☒	A statement on whether measurements were taken from distinct samples or whether the same sample was measured repeatedly
☐	☒	The statistical test(s) used AND whether they are one- or two-sided<br>*Only common tests should be described solely by name; describe more complex techniques in the Methods section.*
☒	☐	A description of all covariates tested
☐	☒	A description of any assumptions or corrections, such as tests of normality and adjustment for multiple comparisons
☐	☒	A full description of the statistical parameters including central tendency (e.g. means) or other basic estimates (e.g. regression coefficient) AND variation (e.g. standard deviation) or associated estimates of uncertainty (e.g. confidence intervals)
☐	☒	For null hypothesis testing, the test statistic (e.g. F, t, r) with confidence intervals, effect sizes, degrees of freedom and P value noted<br>*Give P values as exact values whenever suitable.*
☒	☐	For Bayesian analysis, information on the choice of priors and Markov chain Monte Carlo settings
☒	☐	For hierarchical and complex designs, identification of the appropriate level for tests and full reporting of outcomes
☐	☒	Estimates of effect sizes (e.g. Cohen's d, Pearson's r), indicating how they were calculated

Our web collection on statistics for biologists contains articles on many of the points above.

Software and code

Policy information about availability of computer code

Data collection	In RNA-seq profiling, Multiquant 3.0.3 software (Sciex) was used to quantify all metabolites.
Data analysis	Image J software (version 1.8.0, https://imagej.nih.gov/ij/) was used to semi-quantify gray values of western blots. Statistical analyses were performed with GraphPad Prism 8.0 (GraphPad Software, La Jolla, CA, USA)

For manuscripts utilizing custom algorithms or software that are central to the research but not yet described in published literature, software must be made available to editors and reviewers. We strongly encourage code deposition in a community repository (e.g. GitHub). See the Nature Portfolio guidelines for submitting code & software for further information.

Data

Policy information about availability of data

All manuscripts must include a data availability statement. This statement should provide the following information, where applicable:

- Accession codes, unique identifiers, or web links for publicly available datasets
- A description of any restrictions on data availability
- For clinical datasets or third party data, please ensure that the statement adheres to our policy

The RNA-seq datasets generated in this study have been deposited in GEO database under accession code GSE266942 and GSE270696. The remaining data generated in this study are available in the main text or the supplementary materials. Source data are provided with this paper.

Research involving human participants, their data, or biological material

Policy information about studies with human participants or human data. See also policy information about sex, gender (identity/presentation), and sexual orientation and race, ethnicity and racism.

Reporting on sex and gender	We used human proximal colon randomly from males and females. So these findings applied to either sex. The specific informations of donors were provided in manuscript Supplementary Table 2.
Reporting on race, ethnicity, or other socially relevant groupings	All human participants were Chinese population recruited from First Affiliated Hospital of Kunming Medical University (Kunming, China).
Population characteristics	The specific informations of donors were provided in manuscript Supplementary Table 2.
Recruitment	We informed all related patients of the use of these specimens and got the written informed consent. Healthy biopsy samples were harvested from the proximal colon of healthy individuals or obese patients.
Ethics oversight	This study was approved by The Clinical Research Ethics Committee of Kunming Medical University (No:2017L27).

Note that full information on the approval of the study protocol must also be provided in the manuscript.

Field-specific reporting

Please select the one below that is the best fit for your research. If you are not sure, read the appropriate sections before making your selection.

☒ Life sciences ☐ Behavioural & social sciences ☐ Ecological, evolutionary & environmental sciences

For a reference copy of the document with all sections, see nature.com/documents/nr-reporting-summary-flat.pdf

Life sciences study design

All studies must disclose on these points even when the disclosure is negative.

Sample size	No sample-size calculation was performed. Based on community standards, n=6 or more mice per group was acceptable for animal studies (Nat Commun. 2019;10(1):4971)
Data exclusions	No data were excluded from the analyses
Replication	Generally, each experiment was repeated for three times expect for mice experiments and all attempts at replication were successful with similar results.
Randomization	All samples were randomly allocated among the groups before experiments.
Blinding	Complete blinding is not possible in animal study and cell culture due to different treatment to different groups. We tried to number the mice randomly when collecting tissues and in this way, investigators are blind to the group of one specific sample and can avoid some biases.

Reporting for specific materials, systems and methods

We require information from authors about some types of materials, experimental systems and methods used in many studies. Here, indicate whether each material, system or method listed is relevant to your study. If you are not sure if a list item applies to your research, read the appropriate section before selecting a response.

Materials & experimental systems

n/a	Involved in the study	
☐	☒	Antibodies
☐	☒	Eukaryotic cell lines
☒	☐	Palaeontology and archaeology
☐	☒	Animals and other organisms
☒	☐	Clinical data
☒	☐	Dual use research of concern
☒	☐	Plants

Methods

n/a	Involved in the study	
☒	☐	ChIP-seq
☐	☒	Flow cytometry
☒	☐	MRI-based neuroimaging

Antibodies

Antibodies used	Rabbit anti-B3galt5 (Abclonal, custom-made, Cat#E9390, 1:1000); Rabbit anti-B3galt5 (Sigma-Alrich, Cat#SAB1302633, 1:1000); Rabbit anti-p-Akt (Ser473) (Santa Cruz, Cat#Sc-7985, 1:1000); Rabbit anti-Akt (Boster Biological Technology, Cat#BM4400, 1:1000);

	Rabbit anti-Muc2 (Invitrogen, Cat#30EE6A15, 1:1000); Mouse anti-PXR (Santa Cruz, Cat#sc-48340, 1:200); Mouse anti-beta-catenin (Santa Cruz, Cat#sc-7963, 1:200); Rabbit anti-Ucp-1 (Abcam, Cat#ab10983, 1:1000); Rabbit anti-Pgc-1alpha (Santa Cruz, Cat#sc-517380, 1:1000); Rabbit anti-p-HSL (Cell Signaling Technology, Cat#4126, 1:1000); Rabbit anti-HSL (Santa Cruz, Cat#sc-25843, 1:1000); Rabbit p-Perilipin (Cell Signaling Technology, Cat#100G7E, 1:1000); Rabbit anti-Perilipin (Cell Signaling Technology, Cat#9349, 1:1000); Rabbit Anti-p-ATGL (Abcam, Cat#ab135093, 1:1000); Rabbit anti-ATGL (Cell Signaling Technology, Cat#2138s, 1:1000); Anti-CD16/32 (BD Bioscience, Cat#553141, 1:500); Anti-CD45-BV711 (eBioscience, Cat#407-0451-82, 1:200); Anti-CD11b-FITC (BD Bioscience, Cat#561688, 1:200); Anti-CD206-PE/Cy7 (Biolegend, Cat#141719, 1:200); Anti-CD11c-APC (BD Bioscience, Cat#561119, 1:200); Rabbit IgG2b kappa Isotype Control (eB149/10H5) (eBioscience, Cat#407-4031-81, 1:200); FITC Rat IgG2b, κ Isotype Control (BD Bioscience, Cat#553988, 1:200); PE/Cyanine7 Rat IgG2a, κ Isotype Ctrl (Biolegend, Cat#400521, 1:200); APC Hamster IgG1, λ1 Isotype Control (BD Bioscience, Cat#553956, 1:200); Rat IgG2a kappa Isotype Control (eBR2a), PE (eBioscience, Cat#12-4321-80, 1:200); Anti-SSEA3-FITC (BD Bioscience, Cat#560236, 1:200); Anti-F4/80-PE (eBioscience, Cat#12-4801-80, 1:200); Rat anti-F4/80 (abcam, Cat#ab6640, 1:200); Mouse anti-beta-tubulin (Zen Bio Science, Cat#200608, 1:4000); Goat anti-Rabbit (Licor, Cat#966-32210, 1:20000); Goat anti-Mouse (Licor, Cat#966-32211, 1:20000)
Validation	Antibodies used were commercially available and were validated in multiple previous studies.<br>Rabbit anti-B3galt5(Abclonal) reacts with mouse species, and the applications include WB and ICC/IF;<br>Rabbit anti-B3galt5(Sigma) reacts with human species, and the applications include WB;<br>Rabbit anti-Akt and anti-p-Akt reacts with mouse, rat and human species, and the applications include WB, IHC, ICC/IF and IP;<br>Rabbit anti-Muc2 reacts with mouse and human species, and the applications include WB and ICC/IF;<br>Mouse anti-PXR reacts with mouse and human species, and the applications include WB and IHC;<br>Mouse anti-beta-catenin reacts with mouse, rat and human species, and the applications include WB, IHC, ICC/IF and Elisa;<br>Rabbit anti-Ucp-1 reacts with mouse and Rat species, and the applications include WB and IHC;<br>Rabbit anti-Pgc-1alpha reacts with human species but also reacts with mouse species by validation in the current study, and the antibodies were used for WB;<br>Rabbit anti-p-HSL reacts with mouse and Rat species, and the applications include WB;<br>Rabbit anti-HSL reacts with mouse and Human species, and the applications include WB, ICC/IF and IP;<br>Rabbit p-Perilipin react with mouse species, and was used for WB;<br>Rabbit anti-Perilipin reacts with mouse and Human species, and the applications include WB,IHC, ICC/IF and IP;<br>Rabbit Anti-p-ATGL reacts with mouse species, and the applications include WB and Elisa;<br>Rabbit Anti-ATGL reacts with mouse and Human species, and the applications include WB, ICC/IF and IP;<br>Rat Anti-CD45-BV711 reacts with mouse species, and the applications include Flow Cyt;<br>Rat Anti-CD11b-FITC reacts with mouse species, and the applications include Flow Cyt and ICFC;<br>Rat Anti-CD206-PE/Cy7 reacts with mouse and Human species, and the applications include Flow Cyt and ICC/IF;<br>Hamster Anti-CD11c-APC reacts with mouse species, and the applications include Flow Cyt;<br>Rat Anti-F4/80-PE reacts with mouse and Human species, and the applications include Flow Cyt, IHC and ICC/IF;<br>Rat anti-F4/80 reacts with mouse species, and the applications include Flow Cyt and ICC/IF;<br>Rat Anti-SSEA3-FITC reacts with mouse and Human species, and the applications include Flow Cyt;<br>Mouse anti-beta-tubulin reacts with mouse, Human, Monkey, Goat, Rat, and Hamster species, and the applications include WB and ICC/IF;

Eukaryotic cell lines

Policy information about cell lines and Sex and Gender in Research

Cell line source(s)	Human LS174T cells (ATCC, Cat#CL-188, isolated from the colon of a White, 58-year-old, female adenocarcinoma patient with colorectal cancer) and human embryonic kidney 293 (HEK293; ATCC, Cat#CRL-1573, isolated from the kidney of a human embryo)
Authentication	The cell line used was authenticated by STR profiling
Mycoplasma contamination	The cell line was tested negative for mycoplasma contamination.
Commonly misidentified lines<br>(See ICLAC register)	No misidentified line was used in the study

Animals and other research organisms

Policy information about studies involving animals; ARRIVE guidelines recommended for reporting animal research, and Sex and Gender in Research

Laboratory animals	8-week-old male Ob/ob mice and C57BL/6J mice were provided by Beijing HFK Bioscience (Beijing, China). 8-week-old male PXR knockout (PXR-/-) mice on C57BL/6J background were obtained as a gift from Capital Medical University (Beijing, China). 8-week-old male B3galt5 knockout mice (B3galt5-/-), B3galt5-floxed (B3galt5f/f) mice and VillinCre-ERT mice on C57BL/6J background were purchased from Beijing Biocytogen Pharmaceuticals Co., Ltd (Beijing, China).B3galt5f/f mice were interbred with VillinCre-ERT mice to generate 8-week-old male intestine-specific B3galt5-deficient mice (B3galt5△IEC).Mice were group-housed in individually ventilated cages under controlled temperature and humidity with a 12-h light–dark cycle.
Wild animals	The study did not involve wild animals.
Reporting on sex	Only male C57BL/6J mice were used because female mice are not susceptible to diet-induced obesity and metabolic disorders, such as insulin resistance and hypertriglyceridemia.

Field-collected samples	The study did not involve samples collected from the field.
Ethics oversight	All animal protocols were approved by Sichuan University Animal Care and Use Committee.

Note that full information on the approval of the study protocol must also be provided in the manuscript.

Plants

Seed stocks	The study did not involve plants.
Novel plant genotypes	not applicable
Authentication	not applicable

Flow Cytometry

Plots

Confirm that:

- [x] The axis labels state the marker and fluorochrome used (e.g. CD4-FITC).
- [x] The axis scales are clearly visible. Include numbers along axes only for bottom left plot of group (a 'group' is an analysis of identical markers).
- [x] All plots are contour plots with outliers or pseudocolor plots.
- [x] A numerical value for number of cells or percentage (with statistics) is provided.

Methodology

Sample preparation	Stromal vascular fraction (SVF) from epidydimal white adipose tissue (eWAT) was isolated from 12-week HFD feeding male C57BL/6J, B3galt5 knockout and B3galt5△IEC mice with collagenase I (Signa-Aldrich) for 20 min at 37℃. The suspension was filtered with 150 mesh filter and then centrifuged at 500g, for 5 min at 4℃ to remove adipocyte from SVF. The SVF was then incubated with red blood cell lysis buffer (Beyotime, C3702) for 5 min at room temperature. SVF was resuspended in PBS containing 1mM EDTA, 25 mM HEPES, and 1%FBS after centrifugation. After incubating with Fc-block anti-CD16/32 for 10 min at 4℃, SVF was then stained with fluorescence-labeled primary antibodies for 30min on ice. Before analysis, SVF was incubated with 7-AminoactinoMycin D for 10 min at room temperature to exclude the apoptosis cells. After excluding non-specific staining and adjusting fluorescence compensation by staining cells with single fluorescence-labeled primary antibodies
Instrument	The SVF stained with mix antibodies was collected and analyzed by CytoFlex (BD FACSAria™ Fusion, USA).
Software	FlowJo™ software (version 10, https://www.bdbiosciences.com/zh-cn/products/software/flowjo-v10-software) was used to analyze the flow cytometry data.
Cell population abundance	The purity of the post-sorted cells was more than 95% as verified by flow cytometry.
Gating strategy	The gating strategy was shown in Supplementary Figure 6a. Generally, single cell gates based on FSC-A and FSC-H, and SSC-A and SSC-H were used to exclude non-singlets. The relative isotype control was utilized to identify positive cells.

- [x] Tick this box to confirm that a figure exemplifying the gating strategy is provided in the Supplementary Information.
