# Supplementary material for: B3galt5 functions as a PXR target gene and regulates obesity and insulin resistance by maintaining intestinal integrity
Source: Nat Commun. 2024 Jul 14;15:5919. doi: 10.1038/s41467-024-50198-z (PMC11247088; doi:10.1038/s41467-024-50198-z)
Supplement: Supplementary file 4 — Source Data [file 41467_2024_50198_MOESM4_ESM.zip › source data/Uncropped blots.pptx]

## Slide 1
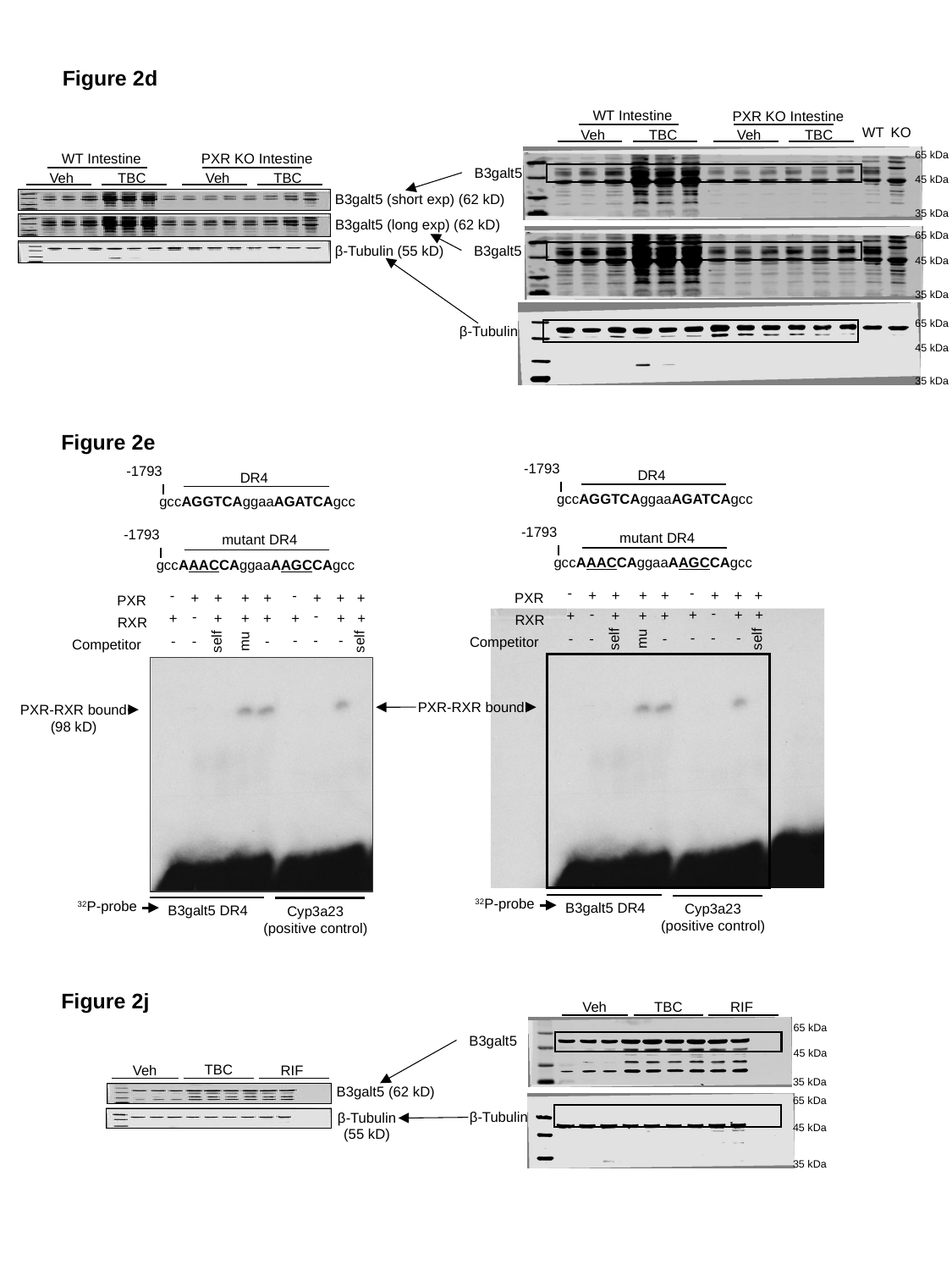

Figure 2d
WT Intestine
PXR KO Intestine
KO
WT
Veh
TBC
Veh
TBC
65 kDa
WT Intestine
PXR KO Intestine
B3galt5
Veh
TBC
Veh
TBC
45 kDa
B3galt5 (short exp) (62 kD)
35 kDa
B3galt5 (long exp) (62 kD)
65 kDa
B3galt5
β-Tubulin (55 kD)
45 kDa
35 kDa
65 kDa
β-Tubulin
45 kDa
35 kDa
Figure 2e
-1793
-1793
DR4
DR4
gccAGGTCAggaaAGATCAgcc
gccAGGTCAggaaAGATCAgcc
-1793
-1793
mutant DR4
mutant DR4
gccAAACCAggaaAAGCCAgcc
gccAAACCAggaaAAGCCAgcc
-
-
+
+
+
+
+
+
+
PXR
-
-
+
+
+
+
+
+
+
RXR
mu
self
-
-
-
self
-
-
-
Competitor
32P-probe
 B3galt5 DR4
Cyp3a23
(positive control)
-
-
+
+
+
+
+
+
+
PXR
-
-
+
+
+
+
+
+
+
RXR
mu
self
-
-
-
self
-
-
-
Competitor
32P-probe
 B3galt5 DR4
Cyp3a23
(positive control)
PXR-RXR bound
PXR-RXR bound
(98 kD)
Figure 2j
TBC
RIF
Veh
65 kDa
B3galt5
45 kDa
TBC
RIF
Veh
35 kDa
B3galt5 (62 kD)
65 kDa
β-Tubulin
β-Tubulin
(55 kD)
45 kDa
35 kDa

## Slide 2
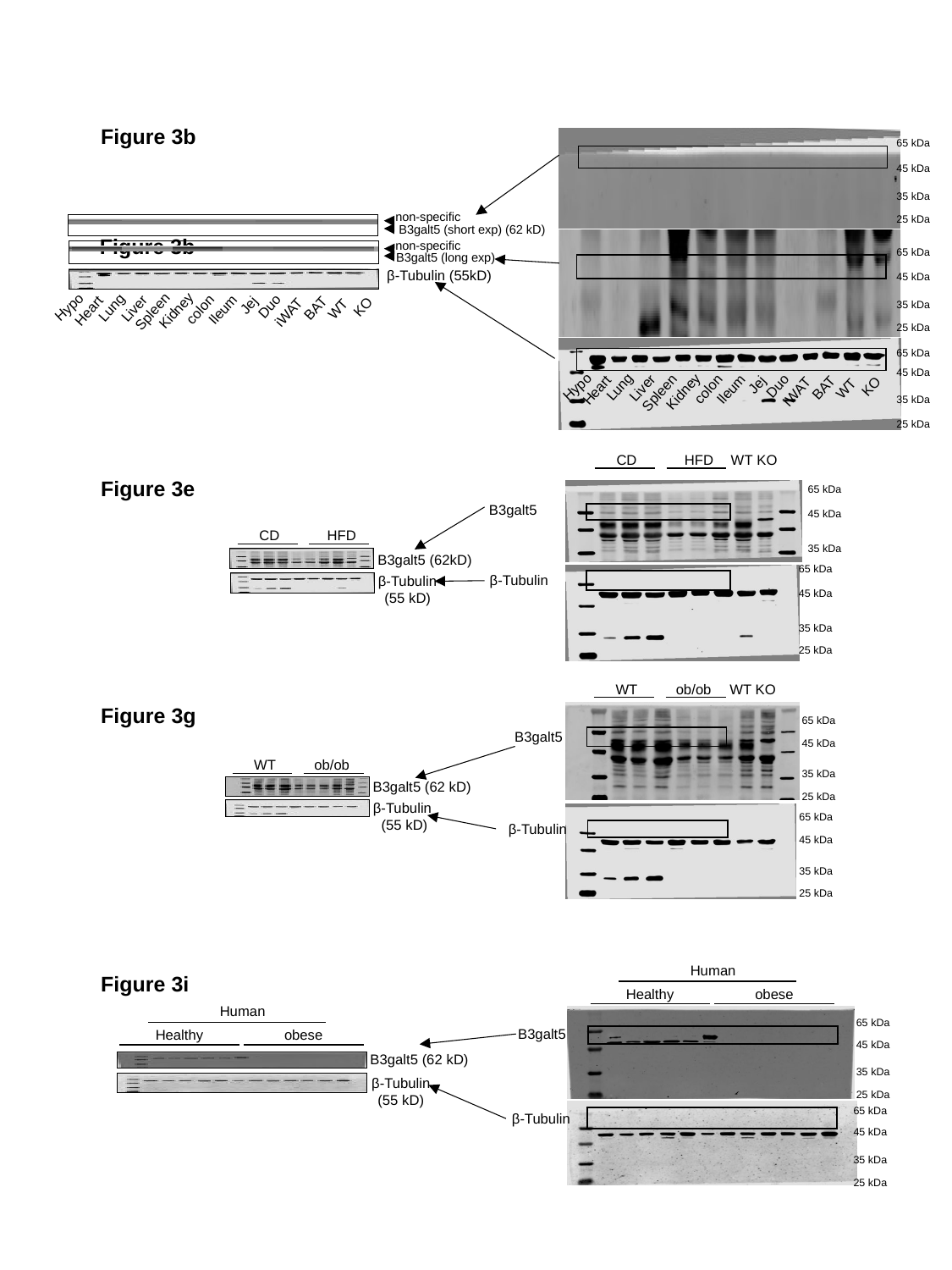

Figure 3b
65 kDa
45 kDa
35 kDa
non-specific
25 kDa
B3galt5 (short exp) (62 kD)
Figure 3b
non-specific
65 kDa
B3galt5 (long exp)
β-Tubulin (55kD)
45 kDa
WT
BAT
Jej
Hypo
Duo
Lung
Liver
KO
iWAT
35 kDa
colon
Ileum
Kidney
Heart
Spleen
25 kDa
65 kDa
45 kDa
WT
BAT
Jej
Hypo
Duo
Lung
Liver
KO
iWAT
colon
Ileum
Heart
Kidney
Spleen
35 kDa
25 kDa
CD
HFD
WT
KO
Figure 3e
65 kDa
B3galt5
45 kDa
CD
HFD
B3galt5 (62kD)
β-Tubulin
(55 kD)
35 kDa
65 kDa
β-Tubulin
45 kDa
35 kDa
25 kDa
WT
ob/ob
WT
KO
Figure 3g
65 kDa
B3galt5
45 kDa
WT
ob/ob
B3galt5 (62 kD)
β-Tubulin
(55 kD)
35 kDa
25 kDa
65 kDa
β-Tubulin
45 kDa
35 kDa
25 kDa
Human
Figure 3i
Healthy
obese
Human
65 kDa
B3galt5
Healthy
obese
45 kDa
B3galt5 (62 kD)
35 kDa
β-Tubulin
(55 kD)
25 kDa
65 kDa
β-Tubulin
45 kDa
35 kDa
25 kDa

## Slide 3
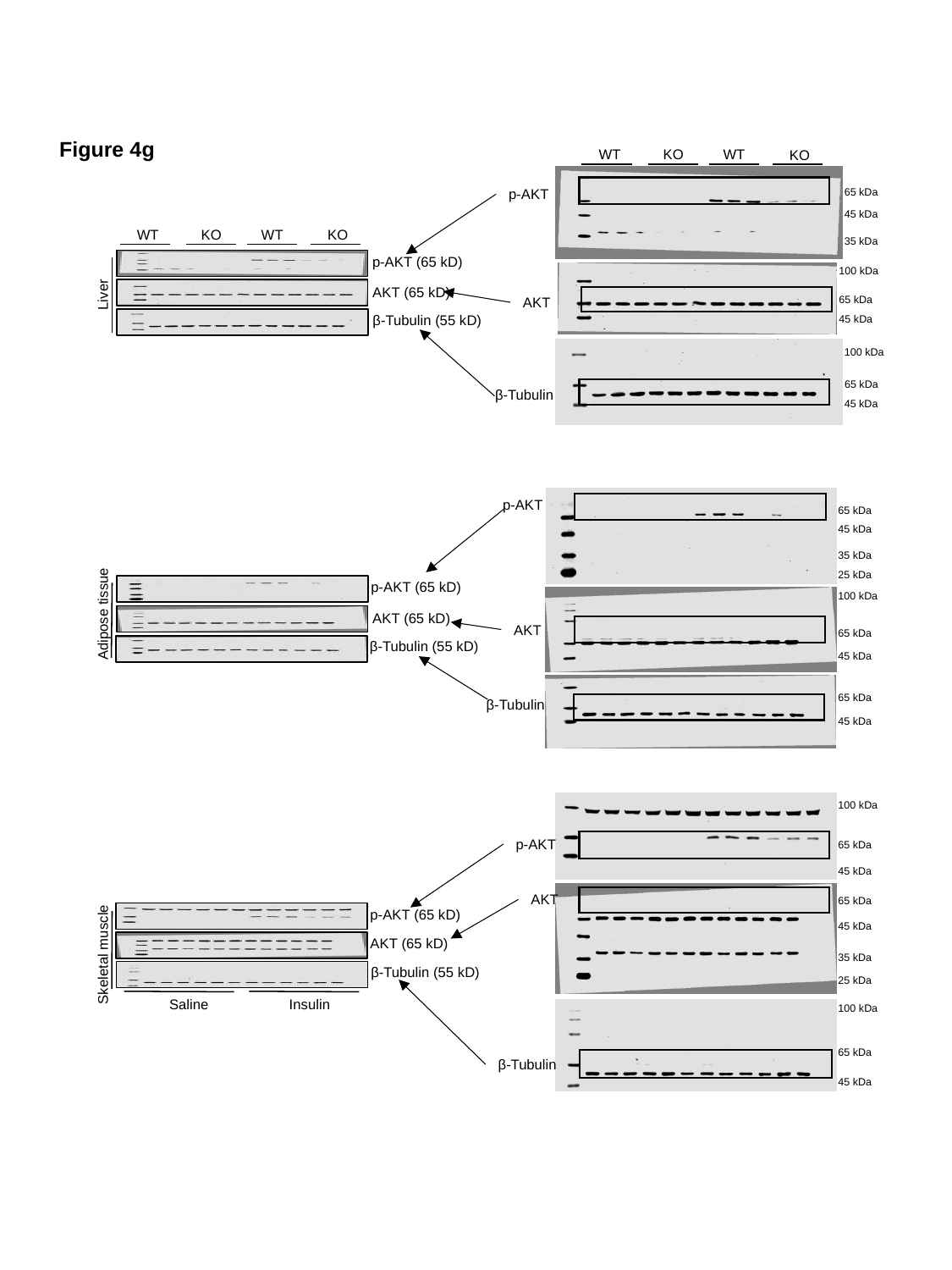

Figure 4g
WT
WT
KO
KO
p-AKT
65 kDa
45 kDa
WT
WT
KO
KO
35 kDa
p-AKT (65 kD)
100 kDa
Liver
AKT (65 kD)
65 kDa
AKT
β-Tubulin (55 kD)
45 kDa
100 kDa
65 kDa
β-Tubulin
45 kDa
p-AKT
65 kDa
45 kDa
35 kDa
25 kDa
p-AKT (65 kD)
100 kDa
Adipose tissue
AKT (65 kD)
AKT
65 kDa
β-Tubulin (55 kD)
45 kDa
65 kDa
β-Tubulin
45 kDa
100 kDa
p-AKT
65 kDa
45 kDa
AKT
65 kDa
p-AKT (65 kD)
45 kDa
AKT (65 kD)
Skeletal muscle
35 kDa
β-Tubulin (55 kD)
25 kDa
Saline
Insulin
100 kDa
65 kDa
β-Tubulin
45 kDa

## Slide 4
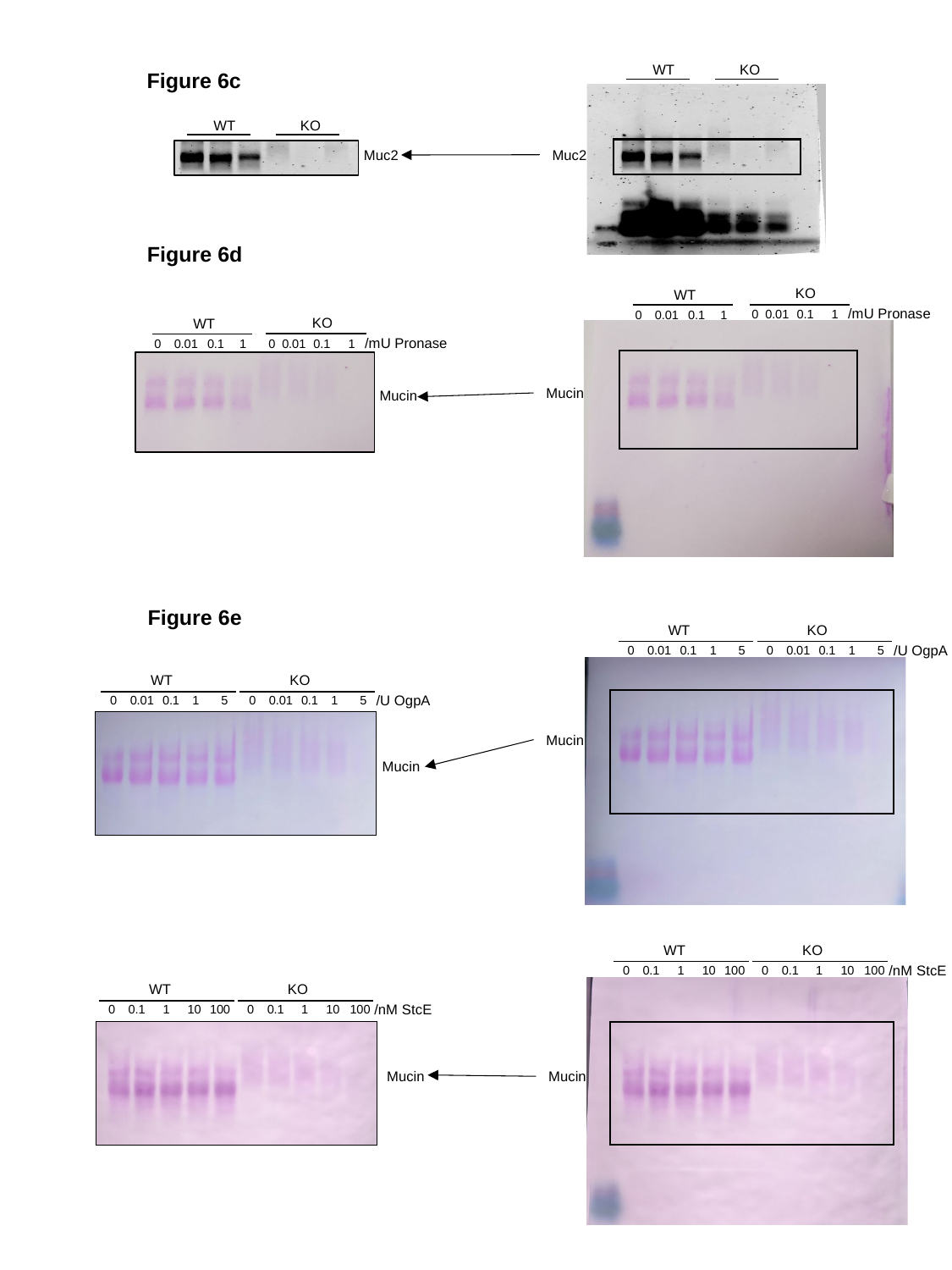

WT
KO
Figure 6c
WT
KO
Muc2
Muc2
Figure 6d
KO
WT
/mU Pronase
0
0.01
0.1
1
0
0.01
0.1
1
KO
WT
/mU Pronase
0
0.01
0.1
1
0
0.01
0.1
1
Mucin
Mucin
Figure 6e
WT
KO
/U OgpA
0
0.01
0.1
1
5
0
0.01
0.1
1
5
WT
KO
/U OgpA
0
0.01
0.1
1
5
0
0.01
0.1
1
5
Mucin
Mucin
WT
KO
/nM StcE
100
10
100
1
0
0.1
1
10
0
0.1
WT
KO
/nM StcE
100
10
100
1
0
0.1
1
10
0
0.1
Mucin
Mucin

## Slide 5
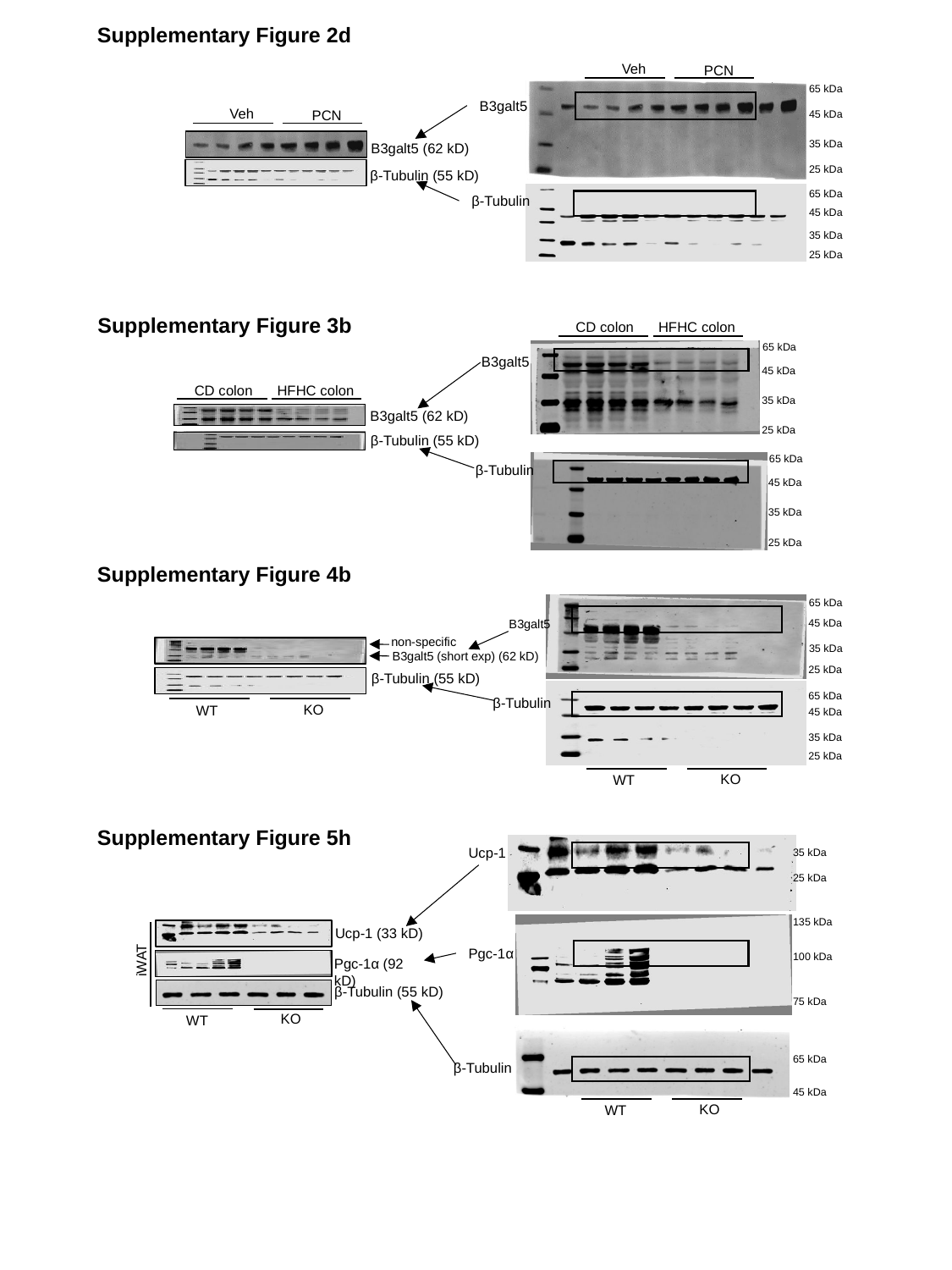

Supplementary Figure 2d
Veh
PCN
65 kDa
B3galt5
Veh
PCN
B3galt5 (62 kD)
β-Tubulin (55 kD)
45 kDa
35 kDa
25 kDa
65 kDa
β-Tubulin
45 kDa
35 kDa
25 kDa
Supplementary Figure 3b
CD colon
HFHC colon
65 kDa
B3galt5
45 kDa
CD colon
HFHC colon
35 kDa
B3galt5 (62 kD)
25 kDa
β-Tubulin (55 kD)
65 kDa
β-Tubulin
45 kDa
35 kDa
25 kDa
Supplementary Figure 4b
65 kDa
B3galt5
45 kDa
non-specific
35 kDa
B3galt5 (short exp) (62 kD)
25 kDa
β-Tubulin (55 kD)
65 kDa
β-Tubulin
KO
WT
45 kDa
35 kDa
25 kDa
KO
WT
Supplementary Figure 5h
Ucp-1
35 kDa
25 kDa
135 kDa
Ucp-1 (33 kD)
iWAT
Pgc-1α (92 kD)
β-Tubulin (55 kD)
KO
WT
Pgc-1α
100 kDa
75 kDa
65 kDa
β-Tubulin
45 kDa
KO
WT

## Slide 6
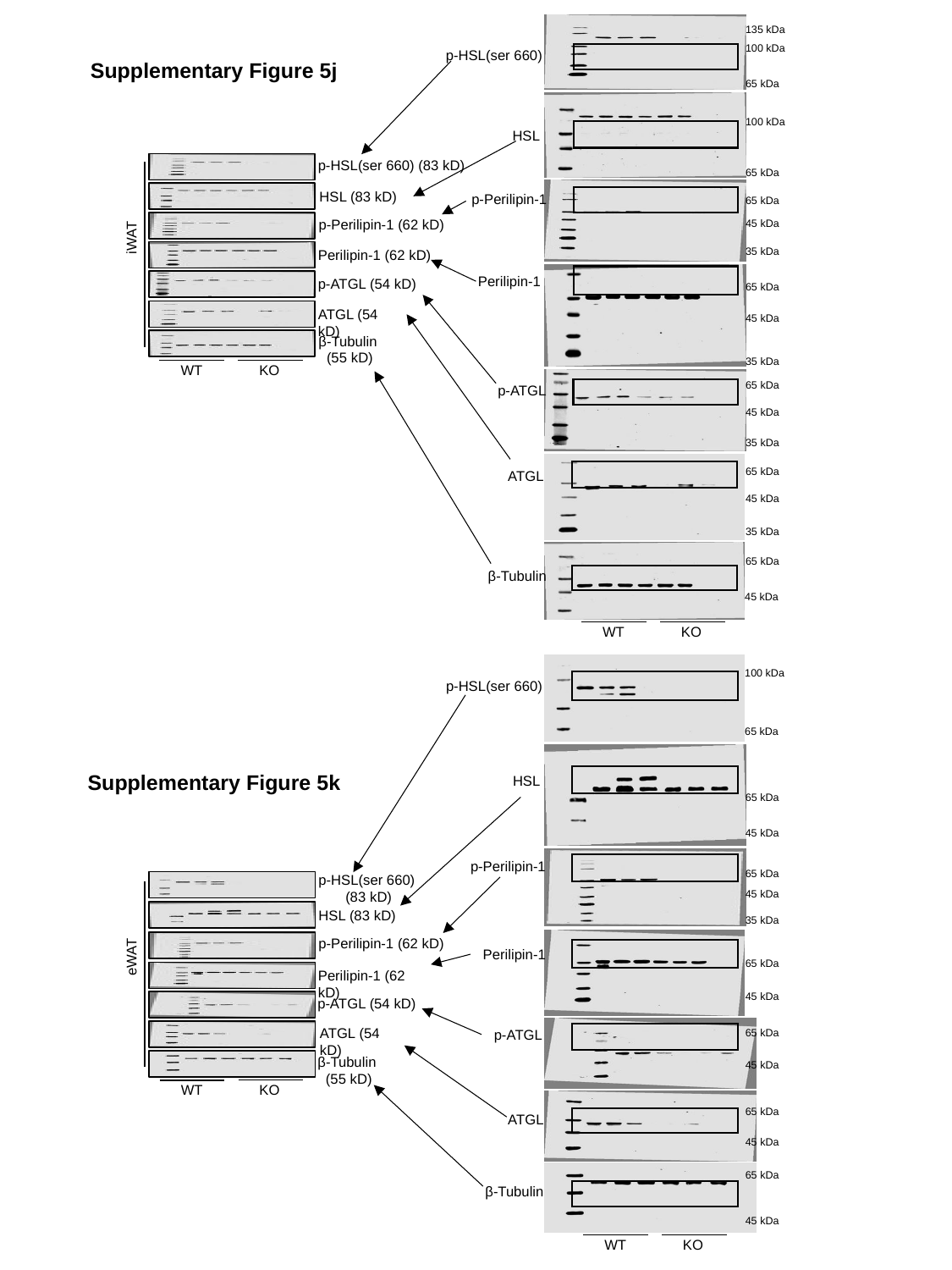

135 kDa
100 kDa
p-HSL(ser 660)
Supplementary Figure 5j
65 kDa
100 kDa
 HSL
p-HSL(ser 660) (83 kD)
 HSL (83 kD)
 p-Perilipin-1 (62 kD)
iWAT
Perilipin-1 (62 kD)
p-ATGL (54 kD)
ATGL (54 kD)
β-Tubulin
(55 kD)
WT
KO
65 kDa
 p-Perilipin-1
65 kDa
45 kDa
35 kDa
Perilipin-1
65 kDa
45 kDa
35 kDa
65 kDa
p-ATGL
45 kDa
35 kDa
65 kDa
ATGL
45 kDa
35 kDa
65 kDa
β-Tubulin
45 kDa
WT
KO
100 kDa
p-HSL(ser 660)
65 kDa
Supplementary Figure 5k
 HSL
65 kDa
45 kDa
p-HSL(ser 660)
(83 kD)
 HSL (83 kD)
 p-Perilipin-1 (62 kD)
eWAT
Perilipin-1 (62 kD)
p-ATGL (54 kD)
ATGL (54 kD)
β-Tubulin
(55 kD)
WT
KO
 p-Perilipin-1
65 kDa
45 kDa
35 kDa
Perilipin-1
65 kDa
45 kDa
65 kDa
p-ATGL
45 kDa
65 kDa
ATGL
45 kDa
65 kDa
β-Tubulin
45 kDa
WT
KO

## Slide 7
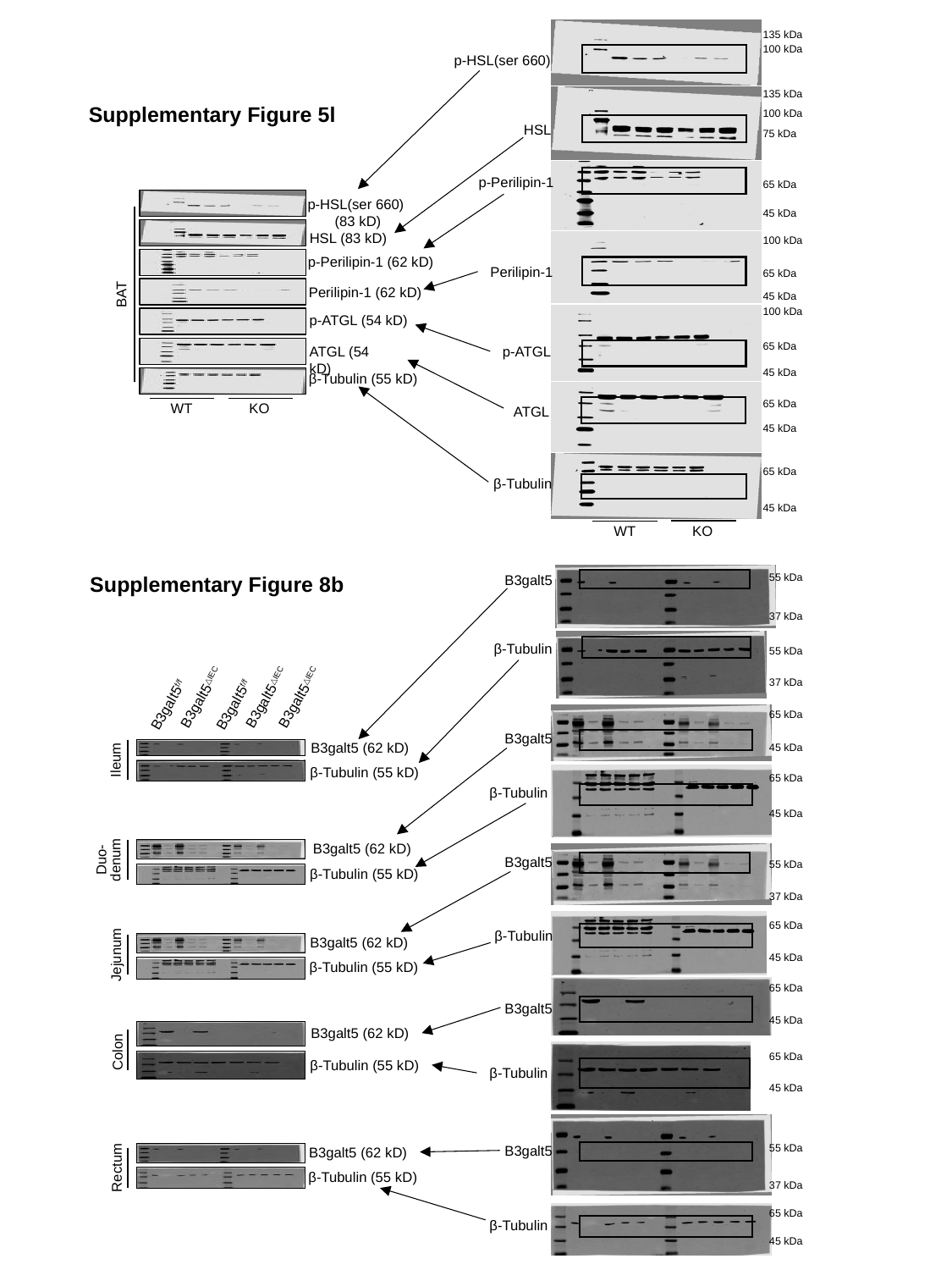

135 kDa
100 kDa
p-HSL(ser 660)
135 kDa
Supplementary Figure 5l
100 kDa
 HSL
75 kDa
 p-Perilipin-1
65 kDa
p-HSL(ser 660)
(83 kD)
 HSL (83 kD)
 p-Perilipin-1 (62 kD)
Perilipin-1 (62 kD)
BAT
p-ATGL (54 kD)
ATGL (54 kD)
β-Tubulin (55 kD)
WT
KO
45 kDa
100 kDa
Perilipin-1
65 kDa
45 kDa
100 kDa
65 kDa
p-ATGL
45 kDa
65 kDa
ATGL
45 kDa
65 kDa
β-Tubulin
45 kDa
WT
KO
55 kDa
Supplementary Figure 8b
B3galt5
37 kDa
β-Tubulin
55 kDa
37 kDa
B3galt5△IEC
B3galt5△IEC
B3galt5△IEC
B3galt5f/f
B3galt5f/f
65 kDa
B3galt5
B3galt5 (62 kD)
45 kDa
Ileum
β-Tubulin (55 kD)
65 kDa
β-Tubulin
45 kDa
B3galt5 (62 kD)
Duo-
denum
B3galt5
55 kDa
β-Tubulin (55 kD)
37 kDa
65 kDa
β-Tubulin
B3galt5 (62 kD)
Jejunum
45 kDa
β-Tubulin (55 kD)
65 kDa
B3galt5
45 kDa
B3galt5 (62 kD)
Colon
65 kDa
β-Tubulin (55 kD)
β-Tubulin
45 kDa
55 kDa
B3galt5
B3galt5 (62 kD)
Rectum
β-Tubulin (55 kD)
37 kDa
65 kDa
β-Tubulin
45 kDa
